# Cross-platform comparison of independent datasets identifies an immune signature associated with improved survival in metastatic melanoma

**DOI:** 10.18632/oncotarget.7361

**Published:** 2016-02-13

**Authors:** Ricardo D. Lardone, Seema B. Plaisier, Marian S. Navarrete, Jaime M. Shamonki, John R. Jalas, Peter A. Sieling, Delphine J. Lee

**Affiliations:** ^1^ Department of Translational Immunology, Dirks/Dougherty Laboratory for Cancer Research, John Wayne Cancer Institute, Santa Monica, CA 90404, USA; ^2^ California Cryobank, Los Angeles, CA 90025, USA; ^3^ Department of Pathology at Providence Saint John's Health Center, Santa Monica, CA 90404, USA

**Keywords:** metastatic melanoma, tumor immunology, bioinformatics, rank-rank hypergeometric overlap, B cells

## Abstract

Platform and study differences in prognostic signatures from metastatic melanoma (MM) gene expression reports often hinder consensus arrival. We performed survival/outcome-based pairwise comparisons of three independent MM gene expression profiles using the threshold-free algorithm rank-rank hypergeometric overlap analysis (RRHO). We found statistically significant overlap for genes overexpressed in favorable outcome (FO) groups, but no overlap for poor outcome (PO) groups. This “favorable outcome signature” (FOS) of 228 genes coinciding on all three overlapping gene lists showed immune function predominated in FO MM. Surprisingly, specific cell signature-enrichment analysis showed B cell-associated genes enriched in FO MM, along with T cell-associated genes. Higher levels of B and T cells (p<0.05) and their relative proximity (p<0.05) were detected in FO-to-PO tumor comparisons from an independent MM patients cohort. Finally, expression of FOS in two independent Stage III MM tumor datasets correctly predicted clinical outcome in 12/14 and 44/70 patients using a weighted gene voting classifier (area under the curve values 0.96 and 0.75, respectively). This RRHO-based, cross-study analysis emphasizes the RRHO approach power, confirms T cells relevance for prolonged MM survival, supports a favorable role for B cells in anti-melanoma immunity, and suggests B cells potential as means of intervention in melanoma treatment.

## INTRODUCTION

Melanoma incidence has been increasing for the last 30 years, making it among the fastest growing cancers worldwide [1]. One of its most dangerous features is a high inherent metastatic potential: primary melanomas have up to 1000-times the inherent metastatic potential compared to most other cancers [2]. Before the age of checkpoint blockade, metastatic melanoma (MM) conferred poor median survival rates (<1yr), although some patients survive for many years after their diagnosis of metastatic disease [3]. These MM outcome differences can be due to a combination of diverse biological factors involving the tumor and its relationship to the host, including cells of the immune system [4]. The target for most of the current MM therapeutic approaches advised by the National Comprehensive Cancer Network (NCCN) Guidelines is innate and/or adaptive components of the immune system [5–8]. With the most effective therapy achieving only a response rate of 50% [5], there is still room for improvement [9].

Different gene expression microarray platforms have proliferated since the first array of cDNA fragments appeared in mid-90's [10]. Although the comparison of independent high-throughput gene-expression experiments has proven to be useful to generate hypotheses from gene-expression programs being active in particular biological conditions, a number of relevant variables in each platform still challenge these comparisons [11]. Often, the available methods to compare gene-expression profiles test for correlation, overlap or enrichment between sets of genes [12, 13]. To this end, lists with a continuous range of thousands of gene-expression differences are reduced to just a small fraction of top changing genes by applying thresholds for differential expression, while ignoring genes with small, yet reproducible changes. This problem can be resolved using rank-rank hypergeometric overlap (RRHO) analysis [14]. RRHO is a threshold-free algorithm that can identify an overlapping gene set with greatest statistical significance when comparing two independent high-throughput gene expression profiles. The benefit of a threshold-free approach is that genes with slight but conserved gene expression changes are not excluded from downstream pathway analysis (to this regard, RRHO can be considered a two-dimensional analog of Gene Set Enrichment Analysis [15], another well-known rank-based approach).

A better knowledge of biological factors linked to MM long-term survival will improve therapy and survival We investigated common signatures comparing gene expression profiles of favorable outcome (FO) and poor outcome (PO) MM patients from three independent MM studies. Performing different combinations of RRHO, we found the most conserved gene expression patterns between these three independent MM patient cohorts. A gene signature consistently expressed in FO MM was obtained, from which we performed gene ontology analyses and additional bioinformatics and tissue labeling approaches. Besides inferring the tumor microenvironment status and the potential cell types determining an improved survival, the FO gene signature was also used to predict survival in other MM datasets.

## RESULTS

**Figure 1:**
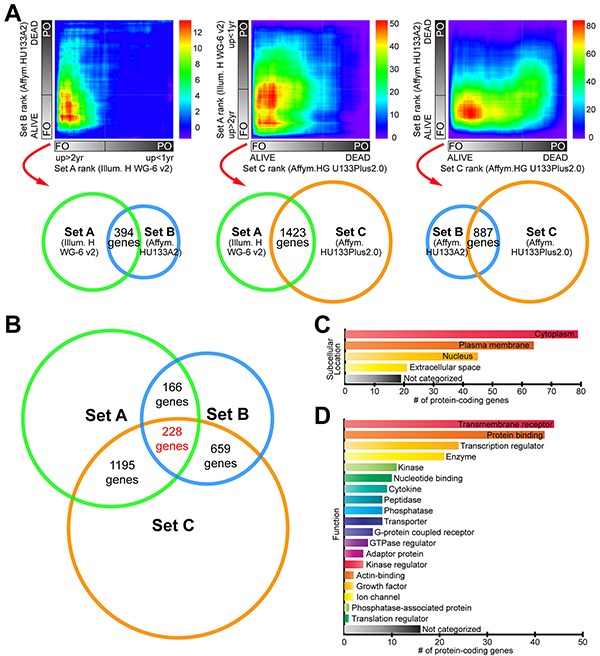
RRHO comparisons of three independent MM gene expression profile datasets identify a “favorable outcome signature” (FOS) **A.** Three complete gene expression profiles were devised by grouping patients from three datasets (“Set A”, “Set B” and “Set C”, see Materials and Methods) into favorable outcome (FO) and poor outcome (PO) groups, ordering genes according to the magnitude and direction of change between survival groups, and compared pairwise using the threshold-free RRHO algorithm. RRHO analysis showed statistically significant overlap between the genes with increased expression in the FO class (see Results). Venn diagrams below RRHO heatmaps represent each pair of datasets compared (with set diameters proportional to correspondent microarray platform sizes) and the corresponding number of overlapping genes. RRHO map signal scale of log_10_-transformed hypergeometric *p*-value is shown on the right of each heatmap. **B.** Venn diagram showing the number of overlapping genes in the FO class in the patient datasets being compared using RRHO analysis. **C.** Summary of the subcellular locations and **D.** functions of the proteins encoded by FOS genes. Supplementary Table S1 contains the full list of 228 overlapping genes common to the three RRHO analyses.

In order to identify novel pathways associated with survival or death in MM, we used RRHO analysis. Three publicly available gene expression datasets from MM studies (GSE22153, “Set A”; GSE46517, “Set B”; GSE19234, “Set C”) were selected and annotated with clinical information for survival/outcome. Criteria used for sample selection are detailed in Materials and Methods section and Supplementary Figure S1. We then performed RRHO analyses on all three possible combinations of datasets, and consistently found a statistically significant overlap in genes exhibiting higher expression in the favorable outcome (FO) MM groups, compared to those with poor outcome or PO (lower left corner of RRHO heatmaps, Figure 1A). These RRHO analyses showed, for example, that genes before rank 1400 in the Set A ranked list and before rank 2100 in the Set B ranked gene list had the most statistically significant number of overlapping genes (n = 394 genes, -log hypergeometric overlap p-value = 19.1). RRHO of Set A and Set C presented 1423 overlapping genes (max -log hypergeometric p-value = 57.6) at the lower left area of map. Meanwhile, RRHO of Set B and Set C datasets exhibited 887 overlapping genes (max -log hypergeometric p-value = 89.1) at the bottom left region of map. Each pair of sets compared and overlapping genes are represented by Venn diagrams below heatmaps (Figure 1A; see legend for details). From these analyses we obtained three lists of overlapping genes having 228 genes in common, thus creating a gene set consistently associated with FO (“favorable outcome signature”, or FOS) across three different studies (Figure 1B and Supplementary Table S1). In contrast to genes from the FO groups, genes from the PO tumors in the three datasets did not exhibit a statistically significant overlap when evaluated using RRHO (Figure 1A). Moreover, dataset pair surveys using a simple cutoff criterion (fold-change greater than 1.5 and a p-value lower than 0.05) found no common genes simultaneously present in all three possible pairings (not shown).

**Figure 2:**
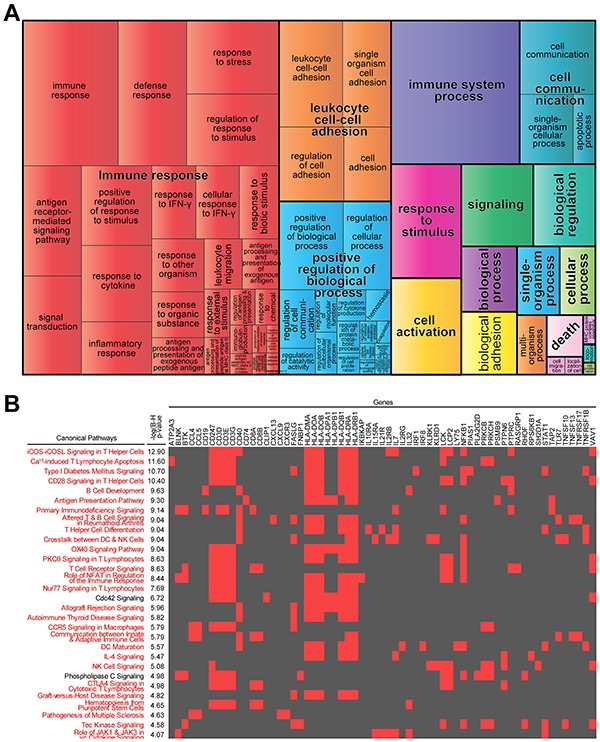
Immune-related biological processes and pathways are enriched in “favorable outcome signature” **A.** Most highly enriched Gene Ontology (GO) terms according to Gene Ontology Consortium and REVIGO algorithms (see Materials and Methods). GO terms are represented by tiles, grouped and colored according to semantic similarities to other GO terms. Tile areas are proportional to −log_10_ *p*-value for each term. **B.** 2-D heatmap view of top-30 canonical pathways and their genes identified by Ingenuity Pathway Analysis. Pathways are ranked by multiple hypothesis corrected *p*-values. Associations between FOS genes and each pathway are indicated with red; immune-related pathways are in red.

Subcellular location for a protein determines its access to interacting partners, enabling their integration into functional biological networks. To determine the biological features of FOS genes relevant for survival in MM, we used Ingenuity Pathway Analysis (IPA) to define their subcellular locations (Figure 1C) and functions (Figure 1D). The multiplicity of locations and functions represented in these FOS genes confers them potential to influence multiple biological processes. This was further supported by gene ontology and pathway analyses using Gene Ontology Consortium database (GO), REduce and VIsualize Gene Ontology (REVIGO) web server and IPA. Strikingly, FOS was enriched in immune-related processes and pathways (Figure 2). GO analysis mapped 161 significantly enriched GO biological functions (B-H p<0.05, Supplementary Table S2). REVIGO analysis of these functions grouped many of them under immune system-related terms like “Immune response”, “leukocyte cell-cell adhesion” and “immune system process” (Figure 2A). Overall, more than 60% of GO terms in FOS were immune, compared to only 4% total in entire GO database. IPA identified 117 significantly enriched canonical pathways (B-H p<0.05, Supplementary Table S3). Almost all (28/30) of the top 30 canonical pathways were immune-related, including numerous immune cell-mediated cytotoxicity mechanisms as well as immune cell activation pathways (Figure 2B and Supplementary Table S3).

**Figure 3:**
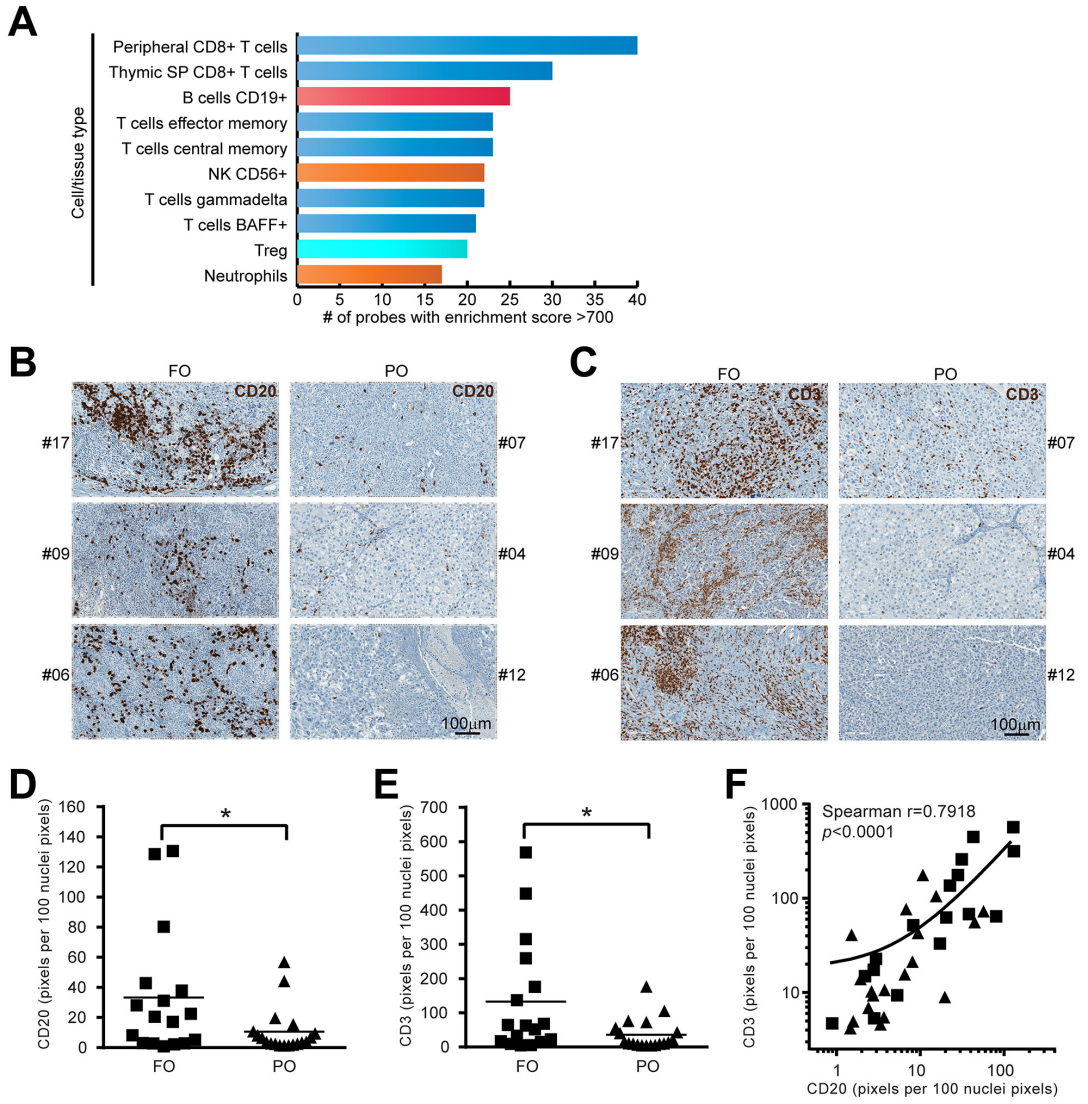
B cells are enriched in FO MM group **A.** Cell type profiling of FOS dataset. The enrichment score (ES) for each FOS probe was assessed in different cell/tissue types contained in “Gene Enrichment Profiler” database (see Materials and Methods). The likelihood of diverse specific cell types infiltrating the tumors was established by ranking the cell/tissue types according to their number of probes with high enrichment score. The top-10 cell/tissue types from the database are shown: T cells (blue), B cells (red), innate effector cells (orange), T_regs_ (light blue). **B.** Representative examples of CD20-IHC labeled FFPE sections from an independent group of MM showing more B cells infiltrates in FO patients compared to PO patients. **C.** Representative examples of CD3-IHC labeled FFPE sections from an independent group of MM showing more T cells infiltrates in FO patients compared to PO patients. **D.** Staining quantification using ImageScope (see Materials and Methods) indicated CD20 label was increased in the FO group (n=36, *p*<0.05). **E.** Staining quantification indicated CD3 label was increased in the FO group (n=36, *p*<0.05). **F.** correlation between CD20 and CD3 levels in MM (n=36, Spearman correlation, *p*<0.0001).

One of the challenges for gene-expression profiling from tumor biopsies is the presence of divergent cell types contributing their mRNA to the total gene expression readout. After finding a positive correlation between the increased expression of immune function-related genes and longer survival, we used the Gene Enrichment Profiler (GEP) database [16] (a curated and publicly available collection of expression intensities converted to enrichment scores) to define the cell types represented in the list of overlapping genes (see Materials and Methods). Figure 3A shows the top-10 cell types displaying the highest number of genes/probes with high enrichment score (>700). Along with genes from other well-known anti-tumor immunity mediators including T cells, B cell-associated genes were enriched in melanoma biopsies from FO patients, suggesting B cells are enriched in FO MM. This was confirmed by immunolabeling of tumor sections from an independent set of MM (Figure 3B). Initial blinded semi-quantitative assessment of labeling by an experienced pathologist (JMS) suggested a trend for increased CD20 labeling on MM from FO patients. Scanning and software-assisted quantification of the labeling indicated higher levels of CD20+ cells on FO patients (p<0.05), supporting a favorable role for B cells in anti-melanoma immunity (Figure 3D). Similar to CD20, levels of the pan T cell marker (CD3) were increased significantly in FO MM (Figure 3C and 3E). Interestingly, Spearman's rank correlation indicated a positive correlation between CD20 and CD3 levels in MM (Figure 3F; r^2^=0.5602; p<0.0001).

**Figure 4:**
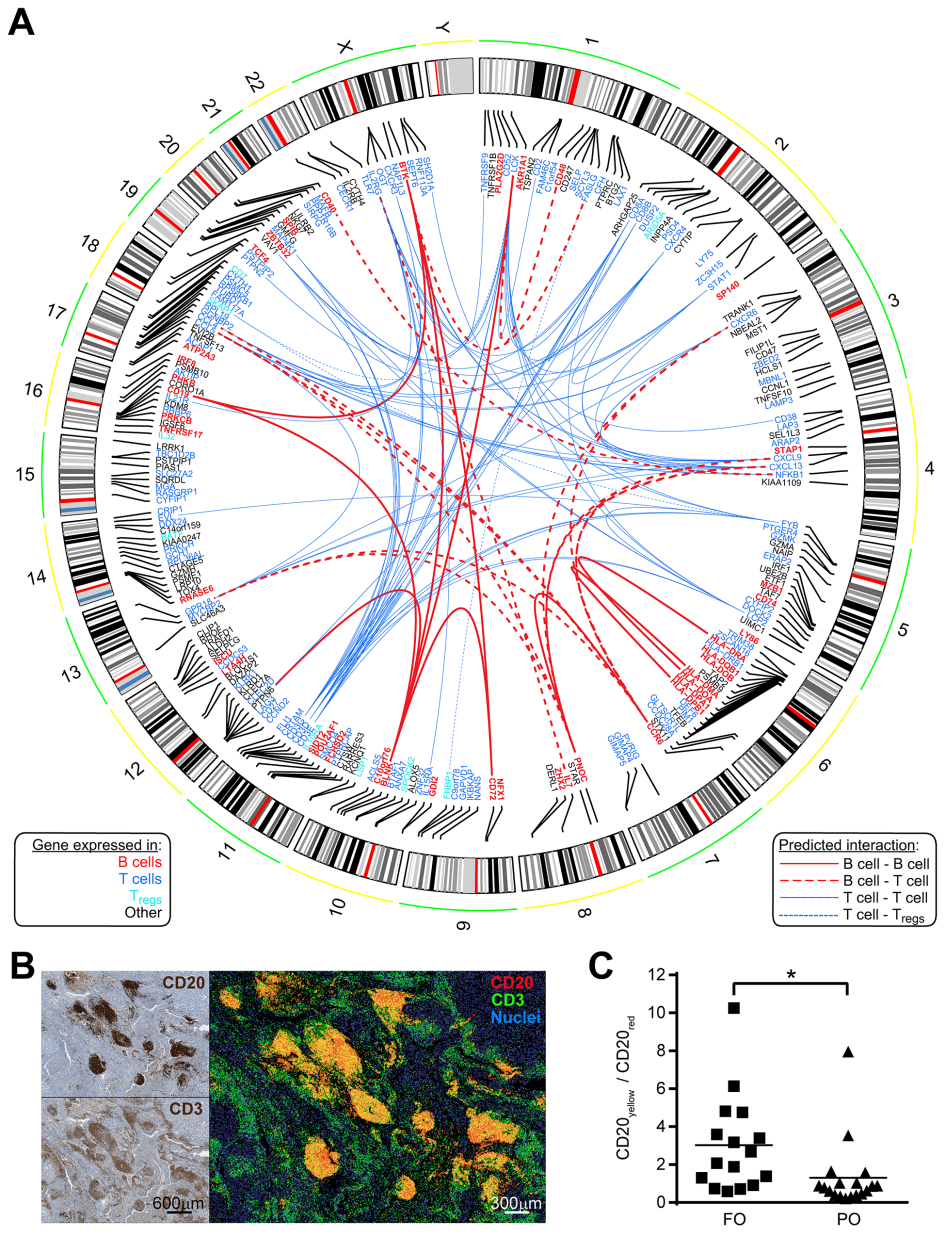
Predicted interactions network from FOS connect cell types in FO MM **A.** Circos plot depicting a network of experimentally observed or high-level-of-confidence predicted interactions was built with integrative information from STRING 9.1 (for interaction evidence) and the Gene Enrichment Profiler (for evidence on cells expressing those nodes/genes) databases. Chromosomal location is shown for each of the 228 overlapping genes. Gene symbols were colored based on the top-10 cell type from Figure 3A showing the highest expression for that gene: B cells (B cells CD19+); T cells (Peripheral CD8+ T cells, Thymic SP CD8+ T cells, T cells effector memory, T cells central memory, γδ T cells, T cells BAFF+); Regulatory T cells (T_regs_). Edges represent curated interactions with experimental evidence or database score higher than 0.7 according to STRING 9.1: B cell-B cell (continuous bold red), B cell- T cells (dashed bold red), T cells- T cells (continuous blue), and T cells- T_regs_ (dotted blue). **B.** B cells show proximity to T cells in MM tissue. Relative proximity of B cells to T cells was revealed by false-color, fluorescent-like image composite (right) of consecutive MM sections individually stained for CD20 and CD3 markers (left). Additive red and green mixing yields yellow in areas of close proximity. **C.** Quantification of CD20_yellow_/CD20_red_ ratio in fluorescent-like image composites of MM sections (using Fiji ImageJ, see Materials and Methods) showing higher ratios in FO patients compared to PO patients (n=34, *p*<0.05).

These findings suggest cooperation between B and T cells in the host response to MM. To identify relationships among B cells and T cells we used the Search Tool for the Retrieval of Interacting Genes/Proteins - STRING (to predict interactions), [13] and the GEP (to predict the cell type best represented by each node). Figure 4A illustrates integrative information from STRING and GEP for the FOS genes, including 15 interactions between “B cells” and “T cells” genes (red dashed edges). We hypothesized that B and T cells would be co-localized to a greater extent in FO *versus* PO tumors. Figure 4B shows a composite example of false-colored consecutive MM sections stained for CD20 (red) and CD3 (green), with areas of close proximity in yellow. Fractions of CD20-labeled cells in close proximity to CD3-labeled cells (expressed as CD20_yellow_/CD20_red_) were higher in FO MM than in PO MM (*p*<0.05, Figure 4C). Using combined bioinformatics approaches and *in situ* immunochemical labeling we find the potential for B and T cell interactions in tumors from FO MM patients.

**Figure 5:**
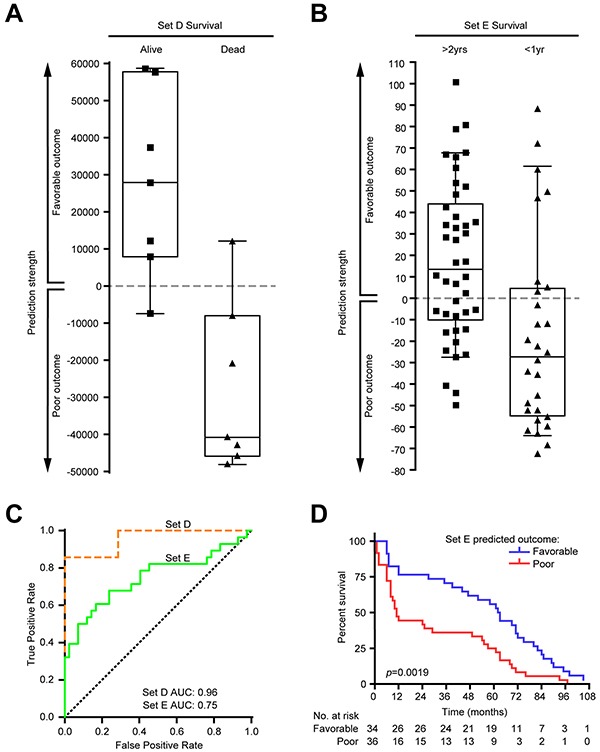
Validation of FOS as outcome predictor in two independent melanoma datasets Weighted gene voting (WGV) using the votes for FOS genes correctly predicted outcome in two independent melanoma stage III datasets. **A.** In Set D (see Materials and Methods), 6/7 (85.7%) patients of favorable outcome and 6/7 (85.7%) patients with poor outcome were correctly predicted. Positive predictive value (PPV): 0.857; negative predictive value (NPV): 0.857; specificity: 0.857; sensitivity: 0.857. **B.** In Set E, 26/42 (61.9%) patients of favorable outcome and 20/28 (71.4%) patients with poor outcome were correctly predicted. PPV: 0.765; NPV: 0.556; specificity: 0.714; sensitivity: 0.619. **C.** Receiver-operating characteristic (ROC) plot showing the performance on both datasets of WGV using FOS genes. Area under the curve (AUC) values are indicated. Dotted diagonal line shows random chance. **D.** Kaplan-Meier curves of survival proportions for Set E patients based on the WGV prediction. Patients with negative class (predicted poor outcome) had significantly reduced survival (*p*=0.0019, log-rank test; survival times correspond to months after tumor resection). Survival information available for Set D patients (“alive” or “dead” by certain date) was insufficient to generate a Kaplan-Meier curve.

Finally, we wanted to evaluate if the expression profile of FOS could predict outcome in MM samples. Toward this effort, a weighted-gene voting (WGV) outcome classifier based on the FOS was built and used to classify stage III MM samples from two independent datasets (Set D and Set E, see Materials and Methods). Outcome was correctly predicted in 12/14 (85.7%) patients from Set D (Figure 5A) and in 46/70 (65.7%) patients from Set E (Figure 5B). In receiver-operating characteristic (ROC) plot, the area under the curve (AUC) values calculated for the performance of WGV were 0.96 for Set D and 0.75 for Set E, indicating FOS has potential for outcome prediction (Figure 5C). Moreover, Kaplan-Meier curves of Set E patients for survival proportions based on the WGV showed that patients scored in the poor outcome (negative) class had significantly reduced survival after tumor resection (*p*=0.0019, log-rank test) compared to those in favorable outcome (positive) class (Figure 5D).

## DISCUSSION

The advent of the “omics” era in the new millennium has brought hope to manage cancer patients using prognostic strategies. However, prognostic signatures derived from gene expression studies in metastatic melanoma (MM) vary from study to study, with no unifying signature across them [17–19]. In the present study we used RRHO, a threshold-free algorithm, to perform pairwise comparisons of three independent MM gene expression profiles. We identified a favorable outcome signature (FOS), a set of genes consistently associated with favorable outcome (FO) MM. Multiple bioinformatics analyses of FOS showed enrichment in immune-related processes and pathways, and inferred the cell types associated with FO MM. Furthermore, increased presence of the primary cell types associated with FO MM was confirmed *in situ* for the pan T cell marker CD3 and the B cell marker CD20, consistent with the work of others for a role of T cells [20], as well as B cells [21] in favorable outcomes. Lastly, this FOS correctly predicted patient outcomes in up to 85% of samples from two independent MM patient datasets.

An association between immune genes expression and improved survival in MM patients has been suggested by previous reports using gene expression microarrays [22, 23]. However, inter-study comparisons for prognosis prediction using standard cutoff criteria (e.g. fold-change > 1.5, p-value < 0.05) found no or only a limited number of genes (at best eight) [18, 19], making it difficult to design a predictive test. In the present study, RRHO detected overlap of 228 genes (FOS) in all three pairwise comparisons conducted, demonstrating its power as a hypothesis-generating tool. In addition, the fact that no common pathways were found in poor outcome (PO) MM highlights the fact that while improved survival or outcome depends on a unifying theme–immunity–there is no unifying theme for poor survival. Recent hypotheses postulate a role for the amount of mutations (mutational load) in melanoma progression [24], although the nature of the datasets used in our study did not allow us to assess this contribution to survival. Further analysis of tumor vs. germline whole exome or whole exome sequencing data is warranted to investigate this idea.

The role of immunity in cancer aligns with the studies of William Coley on therapeutic consequences of streptococci bacteria on sarcomas [25]; with the concept of “cancer immunosurveillance” proposed by Burnet and Thomas [26]; and with the current era of successful immune-based therapies. Indeed, the role of T cells has been exhaustively examined in anti-melanoma responses [27] leading to the ongoing revolution in cancer therapy designed to awaken T cell immunity against tumor [28]. Notwithstanding, the involvement of B cells in melanoma immunobiology has been relatively understudied. Using a cell profiling algorithm, we found that B cells were the second highest ranking cell type after (peripheral or thymic single positive) CD8 T cells. Although there were no probes to measure the pan B cell marker CD20 in any of the dataset platforms (and therefore no CD20 listed in FOS), CD19 (another well-established B cell marker) was indeed present in FOS.

Some reports indicate a negative role for B cells in melanoma [29]. However, B cells are prominent players in autoimmunity and tissue rejection [30], implying their role in breaking tolerance, a key event in successful anti-tumor immunity [31]. Consistent with these findings, animal models of B cell deficiency show more aggressive growth of B16F10 melanoma, which can be ameliorated with adoptive B cell transfer [32, 33]. In these models, B cells seem to promote T cell infiltration and cytokine production within the tumor. Interestingly, there is evidence for an association between Rituximab, an anti-CD20 monoclonal antibody used to treat human B-cell lymphomas or collagen vascular diseases, with more aggressive melanoma forms [34]. A positive role for tumor-infiltrating B cells in the antitumor immune response is also supported by a correlation between low numbers of CD20+ B lymphocytes and primary cutaneous melanoma progression [35]. Thus, our analysis of transcriptional data from multiple datasets supports earlier studies of animal models and primary stage melanomas to suggest a positive role for B cells in metastatic melanomas.

CD40-activated B cells pulsed with melanoma cell lysates potently stimulate peripheral autologous T cells specific to melanoma-associated antigens [36]. B cells can also facilitate expansion of CD8 and CD4 T cells specific for tumor-associated antigens [37], thanks to their ability to present (through MHC class II) or cross-present (through MHC class I) epitopes independent of their B-cell receptor (BCR) specificity [38].

Despite the computational and immunohistochemical evidence of B cells present in FO vs. PO samples, no immunoglobulin light- or immunoglobulin heavy-chain genes were in the FOS. This could be due to limitations of microarray platforms to detect the high variety of immunoglobulin chain genes across the different B cells that underwent diverse VDJC rearrangements. For example, the Illumina human-6 v2.0 expression beadchip platform for Set A lacks probes to detect heavy constant delta, mu, gamma and epsilon chains, as well as light constant kappa and lambda chains. It also has very few probes to cover all combinations of variable regions. Since the RRHO method collapses data to only genes present on all platforms, it removed most probes detecting immunoglobulin genes from the other platforms during analysis. A closer examination of individual datasets indicates immunoglobulin genes measured in Set B FO vs. PO samples showed no statistically significant differences in expression, whereas ~25% of immunoglobulin genes in Set C were comparatively increased in FO vs. PO. However, neither the master regulator for plasma cell driving (“PR domain containing 1” or PRDM1) nor its downstream transcription factor “X-box binding protein 1” (XBP-1, essential for plasma cell differentiation) [39] were enriched in FOS. Although our computational analyses do not allow any clear conclusion about the role of antibodies expressed within tumoral tissue and survival, it is tempting to speculate that the beneficial role for tumor infiltrating B cells in MM may not be through the production of antibodies (i.e. not plasma cells).

Instead, B cells might contribute to antitumor immunity in melanoma by acting as antigen presenting cells (APCs) to T cells. Indeed, FOS contained numerous genes involved in B cell-T cell interactions and their downstream signaling events: Major histocompatibility complex class II (HLA-DMA, HLA-DOA, HLA-DOB, HLA-DPA1, HLA-DPB1, HLA-DQB1, HLA-DRA, HLA-DRB1); T cell receptor/co-receptor (CD3D, CD3E, CD3G, CD8A, CD8B); cytokines/chemokines and their receptors (IL7, IL32, CCL4, CCL5, CXCL9, CXCL13, IL2RB, IL2RG, IL10RA, IL15RA, IL21R, CCR6, CXCR3, CXCR4, CXCR6); accessory and costimulatory molecules (CD74, CD19, CD40); tyrosine kinases (BTK, LCK, LRRK1, MAP4K1, PHKB, PIM1, PRKCB, PRKCH, RPS6KB1, SCYL3, SKAP1); transcription factors (BATF, BTAF1, FLI1, GMFG, IRF1, IRF8, MST1, NFKB1, NFX1, POU2AF1, SPIB, TAF7, TCF4, TFEB, VAV1); and transporters (ATP2A3, FAM117A, SIDT2, SLC27A2, SLC46A3, STAR, STX11, TAP2) (Figure 4A and Table S1). Several stimulatory immune checkpoint genes were associated with FO, either by their presence in FOS (CD27, CD40, IL2RB, TNFRSF9) or by enrichment of their signaling activity pathways (CD28, OX40, ICOS, see Figure 2B). The presence of the activation marker CD38 in FOS would also provide evidence for immune response promotion [40]. With regard to inhibitory immune checkpoint genes, LAG3 was the only inhibitory immune checkpoint molecule associated with the FOS gene signature (Figure 4A and Table S1). Notably, we found no significant difference for the Programmed Death 1 (PD-1) receptor in any of the sets (not shown), and no probe for ligand PD-L1 is present on any of the microarray platforms we studied here. The lack of association of PD-1 with either FO or PO in these datasets suggests the mere expression of this gene at the site of disease does not impact outcome. However, given all the new therapies now available, the expression of various immune checkpoint genes on the tumor or in the tumor microenvironment will likely be much more relevant since they may provide additional targets for therapy.

Human solid tumors often present a complex architecture of immune cells known as tertiary lymphoid structures (TLS), that contribute to antitumor immunity through generation of tumor-controlling primary and /or secondary immune responses [41]. Our data indicated an increased proportion of B cells near T cells in FO MM tumors. In addition, FOS contained genes related to TLS. CXCL13, for example, is among the most potent B cell chemoattractants [42], and its administration can induce and maintain TLS in mouse models [43]. Moreover, the most enriched canonical pathway in FOS was “iCOS-iCOSL signaling in T helper cells” (Figure 2B), which together with expression of *CXCR4* and *IL7* suggests the presence of T follicular helper (T_FH_) cells [44, 45]. T_FH_ and B cells within TLS correlate with survival in breast, colorectal, and lung cancers [42, 46, 47]. Although other T_FH_ cells markers like *BCL6* or *CXCR5* did not appear enriched in FOS, this is reconciled by studies of T cell plasticity suggesting that TH1, TH2 and TH17 cells can substitute for T_FH_ effector functions [48]. All these features can account for the increased presence of B cells in close proximity to T cells in FO MM, supporting their role in antitumor immunity and suggesting the presence of TLS in FO MM tumors.

The present cross-analysis of gene expression microarray sets is applicable to transcriptional studies on other cancers besides melanoma, thereby serving as a discovery engine. The RRHO approach is a powerful tool that unveiled a common signature of genes associated with prolonged MM survival. The FOS successfully predicted patient outcomes, providing a potential decision making tool for treatment of metastatic melanoma. Moreover, this FOS was strongly characterized by immune genes, in particular T and B cells. These findings could have numerous applications for melanoma and other tumors. For example, strategies utilizing gene-modified T cells [49] could include engineering them to express CXCL13, designed to induce B cell recruitment and TLS formation. In addition, targeting vaccine antigen to B cells might enhance current approaches for tumor vaccination therapies. Finally, autologous B cells could facilitate adoptive cell therapy indirectly, by substituting for heterologous APC during T cell expansion, or directly administering them *in vivo* with antitumor T cells.

## MATERIALS AND METHODS

### Gene expression microarray datasets and melanoma specimens

To identify common gene expression profiles, we searched for publicly available gene expression datasets from MM studies annotated with adequate clinical information for survival/outcome, and generated on well-characterized gene expression platforms. Thus, we selected three datasets: “Set A” (GSE22153), “Set B” (GSE46517) and “Set C” (GSE19234). To create uniformity within each dataset, we removed all samples with incomplete clinical information and chose to study samples within one stage per dataset. Thus, for Set A and Set B we kept all Stage IV samples only, whereas for Set C we kept all Stage III samples only. Survival information was used to distribute samples accordingly into favorable outcome (FO) and poor outcome (PO) groups. MM samples from Set A were divided into two groups: <1 year survival and >2 years survival. From published information on metastasis samples in the Set B and Set C studies, we devised analogous sample groups of patients that died from tumor burden and patients that lived at the study endpoint. We also limited our study to those samples that met our predefined FO and PO criteria (Supplementary Figure S1).

Only a minority of the patients from both outcome groups (0% to 30%, depending on the dataset) had received any non-surgical therapy prior to tumor biopsies. Original reports on these datasets (with samples from multiple institutions) described macro-dissection of specimens to ensure a majority of tumor cellularity in all cases. Considering that several samples originated from lymph node metastases, we decided to apply an additional “molecular filter” to avoid bias on gene expression caused by lymphoid tissue. Thus, for each dataset we determined the range of expression levels in the PO groups for each of the six genes/probes most enriched in normal lymph node tissue (as reported by “Gene enrichment profiler database”, see below). We then evaluated these “lymph node classifiers” in the FO samples. Those samples showing expression levels above the established range for three or more classifiers were excluded from analysis. Once sample groups were devised, we created a ranked gene expression profile using log-transformed Student's t-test p-values that were signed either positive for higher average expression in FO sample group, or negative for higher average expression in PO sample group. This placed genes most significantly overexpressed in FO MM patients at the top of the gene list, and genes most significantly overexpressed in the PO MM patients at the bottom of the gene list. The ranked datasets were then subjected to RRHO analysis (see below).

For further validation of the FOS obtained from RRHO, we used gene expression profile and outcome information from two additional MM datasets: “Set D” (GSE46517, only Stage III) and “Set E” (GSE53118). For immunohistochemistry (see below), formalin-fixed, paraffin-embedded (FFPE) tissues of MM lesions surgically resected from short-term and long-term survival MM patients were obtained from Providence-Saint John's Health Center (Santa Monica, CA) under IRB exemption (Study ID LEEDJ-EXPR-08/12, Supplementary Table S4).

### Rank-rank hypergeometric overlap (RRHO)

We compared outcome/survival of MM gene expression profiles using rank-rank hypergeometric overlap (RRHO) analysis [14]. In this algorithm, genes were ranked according to their differential expression between two samples groups, and then these ranked gene expression profiles were iteratively assessed for overlap. The final results were expressed as a heatmap colored by the log-transformed hypergeometric p-value assessing the significance of overlapping genes at each rank threshold pair, such that the highest point on the map identifies the most statistically significant set of overlapping genes. Genes overlapping at this optimal rank threshold pair in all of the three possible pairing combinations were listed (“favorable outcome signature” or FOS) and analyzed further for involvement in specific biological properties and signaling pathways.

### Gene ontology enrichment analysis

Enrichment analysis was performed for the FOS genes using the “Gene Ontology Consortium” enrichment analysis tool [50]. Output list was summarized and visualized using the semantic similarity-based treemap tool of REVIGO (“REduce and VIsualize Gene Ontology” web server) [51]. A *p*-value accounted for the probability that the linking of the FOS list to each GO term was explained by chance alone. GO terms within the same process were grouped and equally colored, and the area values were set proportionally to −log_10_ (multiple hypothesis corrected) *p*-value for each term.

### Ingenuity pathway analysis

The FOS list of genes (with no observation values associated) was uploaded to Ingenuity Pathway Analysis (http://ingenuity.com). The canonical pathways most significantly represented in the FOS set were found using the “Core Analysis” function (pathways were ranked by multiple hypothesis corrected *p*-values).

### Gene enrichment profiler database

To identify the cell subsets and tissues more likely to be represented in the FOS we used the “Gene Enrichment Profiler” database [16]. This tool surveys curated, publicly available gene expression data to retrieve the enrichment scores for each given gene in different cell types and tissues, and has been successfully applied in diverse experimental settings [16, 52, 53]. Probes with enrichment score values >700 were considered to be representative of a cell/tissue type, and a greater number of these probes for the same cell/tissue type, an indication for the presence of that cell type infiltrating the tumor.

### Immunohistochemistry (IHC) of MM tissue

Formalin-fixed, paraffin-embedded (FFPE) tissues of MM lesions surgically resected from short- and long-survival MM patients were obtained under IRB exemption (Study ID LEEDJ-EXPR-08/12). These samples were sectioned at 4 μm intervals, air-dried and deparaffinized by sequentially using xylene and ethanol. Following hydration and high-pH antigen retrieval, sections were blocked with either horse (for IgG2a mouse monoclonal anti-human CD20, clone L26, Dako, Carpinteria, CA) or goat (for polyclonal rabbit anti-human CD3, Dako, Carpinteria, CA) serum. After incubation with primary abs for 1h, the ABC Elite system (Vector Laboratories, Burlingame, CA) for anti-CD20, or HRP-conjugated anti-rabbit IgG (Dako, Carpinteria, CA) for anti-CD3 were used. Detection was achieved by addition of substrate (DAB peroxidase (HRP) substrate kit, Vector Laboratories, Burlingame, CA) for 5 minutes. Slides were counterstained with Mayer's Hematoxylin (Lillie's modification; Dako, Carpinteria, CA) and mounted in Cure Mount II (Electron Microscopy Sciences, Hatfield, PA).

### Image scope

All IHC-stained slides were scanned at an apparent magnification of 20X [resolution of 0.494 μm/pixel (7,970,000 pix/in.)] using the Aperio ScanScope CS and XT systems (Aperio Technologies). The acquired digital images representing whole-tissue sections were viewed and analyzed using “ImageScope analysis software” (version 12; Aperio Technologies, Inc.). Tumor areas were blindly annotated with the assistance of a pathologist (JRJ), and staining was quantitated by applying the “Positive Pixel” algorithm package to IHC and histochemical staining. To allow comparisons between tumor samples of different size, the number of pixels with positive staining for DAB (CD20 or CD3) was expressed as relative to 100 pixels with positive staining for Hematoxylin (nuclei).

### Fiji ImageJ

Whole-tissue sections with positive pixels annotated in ImageScope were exported as TIFF images, and matching sections were carefully aligned and assembled in Adobe Photoshop CS6 to generate pseudo-colored, fluorescent-like composites. Resulting images presented different areas of red (CD20), green (CD3) and yellow (additive red and green mixing). Pixels displaying each color were sequentially selected and counted by changing the “Color Threshold” adjustment in “Fiji ImageJ”, an image-processing package distribution of ImageJ, ImageJ2, Java, Java3D and many plugins [54]. Ratios of measured yellow and red areas were calculated as an indication for the proximity of CD20-labeled cells to CD3-labeled cells.

### STRING 9.1 interaction analyses

The list of FOS genes was uploaded into STRING 9.1 (“Search Tool for the Retrieval of Interacting Genes/Proteins”) database (http:string-db.org) [13] using the “multiple proteins” input form and selecting “Homo sapiens” as the organism. The output network was filtered to show only interactions with experimental evidence and databases scores higher than 0.7.

### Weighted gene voting

We used weighted gene voting [55] to classify tumors from independent datasets as validation for our RRHO derived survival/outcome gene set. Each sample was classified as follows: for each gene in our gene set, we used the signal-to-noise ratio between the two classes as a weight: (mean_alive_ − mean_dead_)/(stdev_alive_ + stdev_dead_). This weight (W) was multiplied to the difference between the expression value in sample being classified (S) and the average of the mean expression values in the two classes: Vote = W (S − (mean_alive_ − mean_dead_)/2). The votes for all 228 genes (FOS) in our survival/outcome gene set were summed to make a prediction: positive sums indicated prediction of “favorable outcome”, while negative sums indicated prediction of “poor outcome”. Furthermore, the sample being predicted was omitted when calculating the mean of the “alive” and “dead” groups for calculating the votes.

### Statistical analyses

All statistical analyses were done using GraphPad Prism software version 6 (GraphPad Software, La Jolla, CA). When appropriate, data were analyzed using the Student's t test (unless otherwise stated). *P*-values lower than 0.05 were considered statistically significant.

## SUPPLEMENTARY MATERIAL FIGURES AND TABLES








