# Supplementary material for: Cross-platform comparison of independent datasets identifies an immune signature associated with improved survival in metastatic melanoma
Source: Oncotarget. 2016 Feb 13;7(12):14415–28. doi: 10.18632/oncotarget.7361 (PMC4924725; doi:10.18632/oncotarget.7361)
Supplement: Supplementary file 1 [file oncotarget-07-14415-s001.pdf]

SUPPLEMENTARY FIGURE TABLES

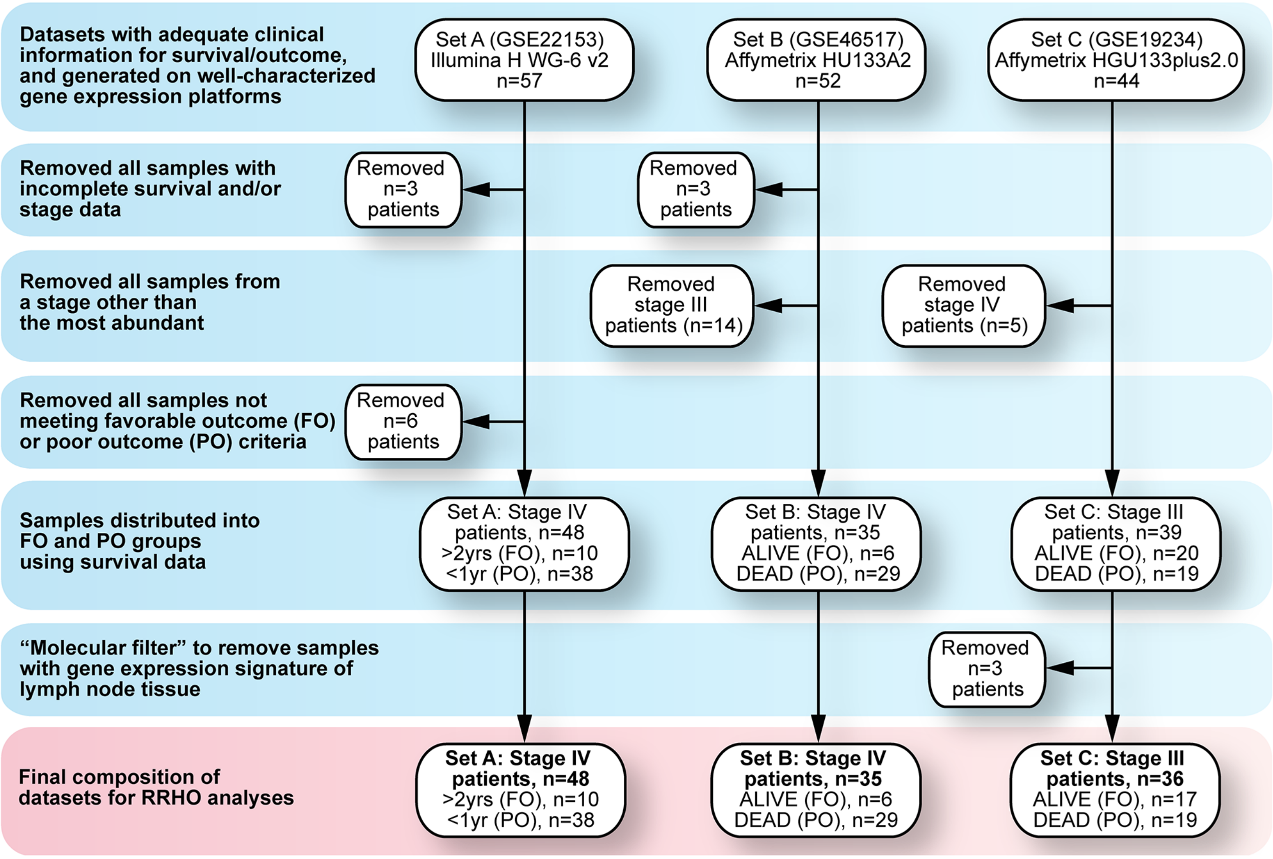

Supplementary Figure 1: Flow diagram for patient datasets analyzed. After initial identification of potential gene expression datasets, we removed samples within each set that were incompletely annotated, belonged to the less abundant stage or that did not meet our predefined FO or PO definitions. Following the grouping into FO and PO categories, samples with a "lymphoid tissue signature" were also filtered out (see Materials and Methods for full details). The final composition of each dataset further used for RRHO analyses is indicated.

Supplementary Table S1: List of 228 optimal overlapping genes present in FO MM groups for all the RRHO analyses. ID (HUGO nomenclature) and Entrez Gene name, subcellular location and type of protein product are detailed for each gene. Cyt: Cytoplasm; Pl Mb: Plasma Membrane; EC Sp: Extracellular Space; Nucl: Nucleus; Unk: Unknown; AP: Adaptor protein; GF: Growth factor; GPCR: G-protein coupled receptor; KR: Kinase regulator; NB: Nucleotide binding; PB: Protein binding; TMR: transmembrane receptor; TR: transcription regulator; NC: not categorized.

See Supplementary File S1

Supplementary Table S2: List of complete GO terms enriched in "favorable outcome signature". Output from Gene Ontology Consortium.

See Supplementary File S2

Supplementary Table S3: Complete list of significant canonical pathways enriched in FOS. Ingenuity Pathway Analysis also identified the FOS molecules for each pathway.

See Supplementary File S3

Supplementary Table S4: Demographic and clinical characteristics of melanoma patients for IHC validation cohort.

Variable	FO	PO	*p*-value ‡
Patients (n=37)	n=17	n=19	
Male (%)	12 (70.6)	15 (78.9)	0.71
Median Age (range)	58 (27-78)	58 (42-77)	0.62 †
Median survival in months (range)	32 (14-68)	6 (0-12)	<0.0001 †
Biopsy location			
Subcutaneous	4 (23.5%)	5 (26.3%)	1
Lung	5 (29.4%)	0	0.02
Intestine	2 (11.8%)	5 (26.3%)	0.41
Brain	5 (29.4%)	4 (21.1%)	0.71
Liver	1 (5.9%)	3 (15.8%)	0.61
Other	0	2 (10.5%)	0.49

‡, *p* values calculated using Fisher's exact test p-Value; †, *p* values for estimations using the non-parametric Mann Whitney test.
