# Supplementary material for: Cross-platform comparison of independent datasets identifies an immune signature associated with improved survival in metastatic melanoma
Source: Oncotarget. 2016 Feb 13;7(12):14415–28. doi: 10.18632/oncotarget.7361 (PMC4924725; doi:10.18632/oncotarget.7361)
Supplement: Supplementary file 2 [file oncotarget-07-14415-s002.docx]

**Table S1: List of 228 optimal overlapping genes present in FO MM groups for all the RRHO analyses.** ID (HUGO nomenclature) and Entrez Gene name, subcellular location and type of protein product are detailed for each gene. Cyt: Cytoplasm; Pl Mb: Plasma Membrane; EC Sp: Extracellular Space; Nucl: Nucleus; Unk: Unknown; AP: Adaptor protein; GF: Growth factor; GPCR: G-protein coupled receptor; KR: Kinase regulator; NB: Nucleotide binding; PB: Protein binding; TMR: transmembrane receptor; TR: transcription regulator; NC: not categorized.

| Symbol | Entrez gene name | Location | Function |
| --- | --- | --- | --- |
| *ACAP1* | ArfGAP with coiled-coil, ankyrin repeat and PH domains 1 | Pl Mb | PB |
| *ACSL5* | acyl-CoA synthetase long-chain family member 5 | Cyt | Enzyme |
| *AKR1A1* | aldo-keto reductase family 1, member A1 (aldehyde reductase) | Cyt | Enzyme |
| *AKTIP* | AKT interacting protein | Cyt | PB |
| *ALDH2* | aldehyde dehydrogenase 2 family (mitochondrial) | Cyt | Enzyme |
| *ALOX5* | arachidonate 5-lipoxygenase | Cyt | Enzyme |
| *ANXA7* | annexin A7 | Pl Mb | Ion channel |
| *ARAP2* | ArfGAP with RhoGAP domain, ankyrin repeat and PH domain 2 | Cyt | GTPase regulator |
| *ARHGAP25* | Rho GTPase activating protein 25 | Cyt | GTPase regulator |
| *ARID5A* | AT rich interactive domain 5A (MRF1-like) | Nucl | TR |
| *ATP2A3* | ATPase, Ca++ transporting, ubiquitous | Cyt | Transporter |
| *BATF* | basic leucine zipper transcription factor, ATF-like | Nucl | TR |
| *BLNK* | B-cell linker | Cyt | AP |
| *BLOC1S1* | biogenesis of lysosomal organelles complex-1, subunit 1 | Cyt | PB |
| *BTAF1* | BTAF1 RNA polymerase II, B-TFIID transcription factor-associated, 170kDa | Nucl | TR |
| *BTG2* | BTG family, member 2 | Nucl | TR |
| *BTK* | Bruton agammaglobulinemia tyrosine kinase | Cyt | Kinase |
| *C10orf76* | chromosome 10 open reading frame 76 | nc | NC |
| *C14orf159* | chromosome 14 open reading frame 159 | Cyt | PB |
| *C1orf54* | chromosome 1 open reading frame 54 | nc | NC |
| *C9orf78* | chromosome 9 open reading frame 78 | nc | NC |
| *CCDC28A* | coiled-coil domain containing 28A | nc | PB |
| *CCDC53* | coiled-coil domain containing 53 | Cyt | PB |
| *CCL4* | chemokine (C-C motif) ligand 4 | EC Sp | Cytokine |
| *CCL5* | chemokine (C-C motif) ligand 5 | EC Sp | Cytokine |
| *CCND2* | cyclin D2 | Nucl | KR |
| *CCNL1* | cyclin L1 | Nucl | KR |
| *CCR6* | chemokine (C-C motif) receptor 6 | Pl Mb | GPCR |
| *CD19* | CD19 molecule | Pl Mb | TMBR |
| *CD2* | CD2 molecule | Pl Mb | TMBR |
| *CD247* | CD247 molecule | Pl Mb | TMBR |
| *CD27* | CD27 molecule | Pl Mb | TMBR |
| *CD38* | CD38 molecule | Pl Mb | Enzyme |
| *CD3D* | CD3d molecule, delta (CD3-TCR complex) | Pl Mb | TMBR |
| *CD3E* | CD3e molecule, epsilon (CD3-TCR complex) | Pl Mb | TMBR |
| *CD3G* | CD3g molecule, gamma (CD3-TCR complex) | Pl Mb | TMBR |
| *CD40* | CD40 molecule, TNF receptor superfamily member 5 | Pl Mb | TMBR |
| *CD47* | CD47 molecule | Pl Mb | TMBR |
| *CD48* | CD48 molecule | Pl Mb | TMBR |
| *CD52* | CD52 molecule | Pl Mb | NC |
| *CD7* | CD7 molecule | Pl Mb | TMBR |
| *CD72* | CD72 molecule | Pl Mb | TMBR |
| *CD74* | CD74 molecule, major histocompatibility complex, class II invariant chain | Pl Mb | TMBR |
| *CD8A* | CD8a molecule | Pl Mb | TMBR |
| *CD8B* | CD8b molecule | Pl Mb | TMBR |
| *CECR1* | cat eye syndrome chromosome region, candidate 1 | EC Sp | Enzyme |
| *CFH* | complement factor H | EC Sp | PB |
| *CLEC2D* | C-type lectin domain family 2, member D | Pl Mb | TMBR |
| *CLEC7A* | C-type lectin domain family 7, member A | Pl Mb | TMBR |
| *CLIP1* | CAP-GLY domain containing linker protein 1 | Cyt | PB |
| *CORO1A* | coronin, actin binding protein, 1A | Cyt | PB |
| *CRIP1* | cysteine-rich protein 1 (intestinal) | Cyt | NB |
| *CRTAM* | cytotoxic and regulatory T cell molecule | Pl Mb | TMBR |
| *CTAGE5* | CTAGE family, member 5 | nc | Enzyme |
| *CTSW* | cathepsin W | Cyt | peptidase |
| *CXCL13* | chemokine (C-X-C motif) ligand 13 | EC Sp | Cytokine |
| *CXCL9* | chemokine (C-X-C motif) ligand 9 | EC Sp | Cytokine |
| *CXCR3* | chemokine (C-X-C motif) receptor 3 | Pl Mb | GPCR |
| *CXCR4* | chemokine (C-X-C motif) receptor 4 | Pl Mb | GPCR |
| *CXCR6* | chemokine (C-X-C motif) receptor 6 | Pl Mb | GPCR |
| *CYFIP1* | Cytic FMR1 interacting protein 1 | Cyt | PB |
| *CYFIP2* | Cytic FMR1 interacting protein 2 | Cyt | PB |
| *CYTH1* | cytohesin 1 | Cyt | PB |
| *CYTH4* | cytohesin 4 | Cyt | PB |
| *CYTIP* | cytohesin 1 interacting protein | Cyt | PB |
| *DAZAP2* | DAZ associated protein 2 | Nucl | PB |
| *DDX24* | DEAD (Asp-Glu-Ala-Asp) box helicase 24 | Nucl | Enzyme |
| *DEF6* | differentially expressed in FDCP 6 homolog (mouse) | EC Sp | PB |
| *DERL1* | derlin 1 | Cyt | PB |
| *DOCK2* | dedicator of cytokinesis 2 | Cyt | PB |
| *DUSP2* | dual specificity phosphatase 2 | Nucl | Phosphatase |
| *ERAP2* | endoplasmic reticulum aminopeptidase 2 | Cyt | peptidase |
| *ETF1* | eukaryotic translation termination factor 1 | Cyt | translation regulator |
| *EVI2B* | ecotropic viral integration site 2B | Pl Mb | NC |
| *EVL* | Enah/Vasp-like | Cyt | PB |
| *FAM117A* | family with sequence similarity 117, member A | nc | Transporter |
| *FAM46C* | family with sequence similarity 46, member C | EC Sp | NC |
| *FASLG* | Fas ligand (TNF superfamily, member 6) | EC Sp | Cytokine |
| *FCHSD2* | FCH and double SH3 domains 2 | nc | PB |
| *FILIP1L* | filamin A interacting protein 1-like | Nucl | NC |
| *FLI1* | Fli-1 proto-oncogene, ETS transcription factor | Nucl | TR |
| *FNBP1* | formin binding protein 1 | Nucl | Enzyme |
| *FYB* | FYN binding protein | Nucl | PB |
| *GAPVD1* | GTPase activating protein and VPS9 domains 1 | Cyt | GTPase regulator |
| *GCH1* | GTP cyclohydrolase 1 | Cyt | Enzyme |
| *GDI2* | GDP dissociation inhibitor 2 | Cyt | PB |
| *GGNBP2* | gametogenetin binding protein 2 | nc | PB |
| *GIMAP4* | GTPase, IMAP family member 4 | Nucl | Phosphatase |
| *GIMAP5* | GTPase, IMAP family member 5 | Cyt | Phosphatase |
| *GLTSCR1L* | GLTSCR1-like | nc | NC |
| *GMFG* | glia maturation factor, gamma | Cyt | GF |
| *GPR18* | G protein-coupled receptor 18 | Pl Mb | GPCR |
| *GZMA* | granzyme A (granzyme 1, cytotoxic T-lymphocyte-associated serine esterase 3) | Cyt | peptidase |
| *GZMH* | granzyme H (cathepsin G-like 2, protein h-CCPX) | Cyt | peptidase |
| *GZMK* | granzyme K (granzyme 3; tryptase II) | Cyt | peptidase |
| *HCLS1* | hematopoietic cell-specific Lyn substrate 1 | Nucl | PB |
| *HLA-DMA* | major histocompatibility complex, class II, DM alpha | Pl Mb | TMBR |
| *HLA-DOA* | major histocompatibility complex, class II, DO alpha | Pl Mb | TMBR |
| *HLA-DOB* | major histocompatibility complex, class II, DO beta | Pl Mb | TMBR |
| *HLA-DPA1* | major histocompatibility complex, class II, DP alpha 1 | Pl Mb | TMBR |
| *HLA-DPB1* | major histocompatibility complex, class II, DP beta 1 | Pl Mb | TMBR |
| *HLA-DQB1* | major histocompatibility complex, class II, DQ beta 1 | Pl Mb | TMBR |
| *HLA-DRA* | major histocompatibility complex, class II, DR alpha | Pl Mb | TMBR |
| *HLA-DRB1* | major histocompatibility complex, class II, DR beta 1 | Pl Mb | TMBR |
| *IGSF6* | immunoglobulin superfamily, member 6 | Pl Mb | TMBR |
| *IKBKAP* | inhibitor of kappa light polypeptide gene enhancer in B-cells, kinase complex-associated protein | Cyt | KR |
| *IL10RA* | interleukin 10 receptor, alpha | Pl Mb | TMBR |
| *IL15RA* | interleukin 15 receptor, alpha | Pl Mb | TMBR |
| *IL21R* | interleukin 21 receptor | Pl Mb | TMBR |
| *IL2RB* | interleukin 2 receptor, beta | Pl Mb | TMBR |
| *IL2RG* | interleukin 2 receptor, gamma | Pl Mb | TMBR |
| *IL32* | interleukin 32 | EC Sp | Cytokine |
| *IL7* | interleukin 7 | EC Sp | Cytokine |
| *INPP4A* | inositol polyphosphate-4-phosphatase, type I, 107kDa | Cyt | Phosphatase |
| *IRF1* | interferon regulatory factor 1 | Nucl | TR |
| *IRF8* | interferon regulatory factor 8 | Nucl | TR |
| *ISCU* | iron-sulfur cluster assembly enzyme | Cyt | PB |
| *KCNQ1* | potassium voltage-gated channel, KQT-like subfamily, member 1 | Pl Mb | Ion channel |
| *KDM8* | lysine (K)-specific demethylase 8 | Nucl | NB |
| *KIAA0247* | KIAA0247 | nc | PB |
| *KIAA1109* | KIAA1109 | nc | PB |
| *KLRK1* | killer cell lectin-like receptor subfamily K, member 1 | Pl Mb | TMBR |
| *KLRD1* | killer cell lectin-like receptor subfamily D, member 1 | Pl Mb | TMBR |
| *LAG3* | lymphocyte-activation gene 3 | Pl Mb | TMBR |
| *LAMP3* | lysosomal-associated membrane protein 3 | Pl Mb | NC |
| *LAP3* | leucine aminopeptidase 3 | Cyt | peptidase |
| *LAX1* | lymphocyte transmembrane adaptor 1 | Cyt | KR |
| *LCK* | lymphocyte-specific protein tyrosine kinase | Cyt | Kinase |
| *LCP2* | lymphocyte cytosolic protein 2 (SH2 domain containing leukocyte protein of 76kDa) | Cyt | AP |
| *LILRB2* | leukocyte immunoglobulin-like receptor, subfamily B (with TM and ITIM domains), member 2 | Pl Mb | TMBR |
| *LRP10* | low density lipoprotein receptor-related protein 10 | Pl Mb | TMBR |
| *LRRK1* | leucine-rich repeat kinase 1 | Cyt | Kinase |
| *LSP1* | lymphocyte-specific protein 1 | Cyt | Actin-binding |
| *LTA4H* | leukotriene A4 hydrolase | Cyt | Enzyme |
| *LY75* | lymphocyte antigen 75 | Pl Mb | TMBR |
| *LY86* | lymphocyte antigen 86 | Pl Mb | NC |
| *MAFB* | v-maf avian musculoaponeurotic fibrosarcoma oncogene homolog B | Nucl | NB |
| *MAP4K1* | mitogen-activated protein kinase kinase kinase kinase 1 | Cyt | Kinase |
| *MBNL1* | muscleblind-like splicing regulator 1 | Nucl | NB |
| *MGA* | MGA, MAX dimerization protein | Nucl | TR |
| *MST1* | macrophage stimulating 1 (hepatocyte growth factor-like) | EC Sp | GF |
| *MYCBP2* | MYC binding protein 2, E3 ubiquitin protein ligase | Nucl | Enzyme |
| *MZB1* | marginal zone B and B1 cell-specific protein | EC Sp | PB |
| *NAIP* | NLR family, apoptosis inhibitory protein | nc | NB |
| *NANS* | N-acetylneuraminic acid synthase | Cyt | Enzyme |
| *NAP1L3* | nucleosome assembly protein 1-like 3 | Nucl | NC |
| *NBEAL2* | neurobeachin-like 2 | Cyt | NC |
| *NFKB1* | nuclear factor of kappa light polypeptide gene enhancer in B-cells 1 | Nucl | TR |
| *NFX1* | nuclear transcription factor, X-box binding 1 | Nucl | TR |
| *NKG7* | natural killer cell group 7 sequence | Pl Mb | PB |
| *OGT* | O-linked N-acetylglucosamine (GlcNAc) transferase | Cyt | Enzyme |
| *PHKB* | phosphorylase kinase, beta | Cyt | Kinase |
| *PIAS1* | protein inhibitor of activated STAT, 1 | Nucl | TR |
| *PIM1* | pim-1 oncogene | Cyt | Kinase |
| *PLA2G2D* | phospholipase A2, group IID | EC Sp | Enzyme |
| *PNOC* | prepronociceptin | EC Sp | PB |
| *POU2AF1* | POU class 2 associating factor 1 | Nucl | TR |
| *PPP1R16B* | protein phosphatase 1, regulatory subunit 16B | Pl Mb | Phosphatase |
| *PRKCB* | protein kinase C, beta | Cyt | Kinase |
| *PRKCH* | protein kinase C, eta | Cyt | Kinase |
| *PSD4* | pleckstrin and Sec7 domain containing 4 | Cyt | NC |
| *PSMB10* | proteasome (prosome, macropain) subunit, beta type, 10 | Cyt | peptidase |
| *PSMB9* | proteasome (prosome, macropain) subunit, beta type, 9 | Cyt | peptidase |
| *PSMC5* | proteasome (prosome, macropain) 26S subunit, ATPase, 5 | Nucl | TR |
| *PSME1* | proteasome (prosome, macropain) activator subunit 1 (PA28 alpha) | Cyt | PB |
| *PSTPIP1* | proline-serine-threonine phosphatase interacting protein 1 | Cyt | Actin-binding |
| *PSTPIP2* | proline-serine-threonine phosphatase interacting protein 2 | Cyt | PB |
| *PTGER4* | prostaglandin E receptor 4 (subtype EP4) | Pl Mb | GPCR |
| *PTPN2* | protein tyrosine phosphatase, non-receptor type 2 | Cyt | Phosphatase |
| *PTPN6* | protein tyrosine phosphatase, non-receptor type 6 | Cyt | Phosphatase |
| *PTPRC* | protein tyrosine phosphatase, receptor type, C | Pl Mb | Phosphatase |
| *PTPRCAP* | protein tyrosine phosphatase, receptor type, C-associated protein | Pl Mb | Phosphatase-associated protein |
| *PVRIG* | poliovirus receptor related immunoglobulin domain containing | nc | NC |
| *RARRES3* | retinoic acid receptor responder (tazarotene induced) 3 | Cyt | Enzyme |
| *RASGRP1* | RAS guanyl releasing protein 1 (calcium and DAG-regulated) | Cyt | GTPase regulator |
| *RBBP6* | retinoblastoma binding protein 6 | Nucl | Enzyme |
| *RHOF* | ras homolog family member F (in filopodia) | Cyt | Enzyme |
| *RNASE6* | ribonuclease, RNase A family, k6 | EC Sp | Enzyme |
| *RNF113A* | ring finger protein 113A | Nucl | PB |
| *RPL19* | ribosomal protein L19 | Cyt | NB |
| *RPL36AL* | ribosomal protein L36a-like | Cyt | PB |
| *RPS6KB1* | ribosomal protein S6 kinase, 70kDa, polypeptide 1 | Cyt | Kinase |
| *SCYL3* | SCY1-like 3 (S. cerevisiae) | Cyt | Kinase |
| *SEL1L3* | sel-1 suppressor of lin-12-like 3 (C. elegans) | nc | NC |
| *SELP* | selectin P (granule membrane protein 140kDa, antigen CD62) | Pl Mb | TMBR |
| *SELPLG* | selectin P ligand | Pl Mb | PB |
| *SEPT6* | septin 6 | Cyt | PB |
| *SH2D1A* | SH2 domain containing 1A | Cyt | AP |
| *SIDT2* | SID1 transmembrane family, member 2 | Cyt | Transporter |
| *SIRPG* | signal-regulatory protein gamma | Pl Mb | TMBR |
| *SKAP1* | src kinase associated phosphoprotein 1 | Cyt | Kinase |
| *SLC27A2* | solute carrier family 27 (fatty acid transporter), member 2 | Cyt | Transporter |
| *SLC46A3* | solute carrier family 46, member 3 | EC Sp | Transporter |
| *SMCO4* | single-pass membrane protein with coiled-coil domains 4 | nc | PB |
| *SP140* | SP140 nuclear body protein | Nucl | TR |
| *SPIB* | Spi-B transcription factor (Spi-1/PU.1 related) | Nucl | TR |
| *SPOCK2* | sparc/osteonectin, cwcv and kazal-like domains proteoglycan (testican) 2 | EC Sp | NC |
| *SQRDL* | sulfide quinone reductase-like (yeast) | Cyt | Enzyme |
| *STAP1* | signal transducing adaptor family member 1 | Cyt | AP |
| *STAR* | steroidogenic acute regulatory protein | Cyt | Transporter |
| *STAT1* | signal transducer and activator of transcription 1, 91kDa | Nucl | TR |
| *STX11* | syntaxin 11 | Pl Mb | Transporter |
| *TAF7* | TAF7 RNA polymerase II, TATA box binding protein (TBP)-associated factor, 55kDa | Nucl | TR |
| *TAP2* | transporter 2, ATP-binding cassette, sub-family B (MDR/TAP) | Cyt | Transporter |
| *TBC1D2B* | TBC1 domain family, member 2B | nc | GTPase regulator |
| *TCF4* | transcription factor 4 | Nucl | TR |
| *TFEB* | transcription factor EB | Nucl | TR |
| *TLR7* | toll-like receptor 7 | Pl Mb | TMBR |
| *TNFRSF17* | tumor necrosis factor receptor superfamily, member 17 | Pl Mb | TMBR |
| *TNFRSF1B* | tumor necrosis factor receptor superfamily, member 1B | Pl Mb | TMBR |
| *TNFRSF9* | tumor necrosis factor receptor superfamily, member 9 | Pl Mb | TMBR |
| *TNFSF10* | tumor necrosis factor (ligand) superfamily, member 10 | EC Sp | Cytokine |
| *TNFSF13* | tumor necrosis factor (ligand) superfamily, member 13 | EC Sp | Cytokine |
| *TOX4* | TOX high mobility group box family member 4 | Nucl | NB |
| *TRAFD1* | TRAF-type zinc finger domain containing 1 | nc | PB |
| *TRANK1* | tetratricopeptide repeat and ankyrin repeat containing 1 | Nucl | TR |
| *TRIM38* | tripartite motif containing 38 | nc | PB |
| *TSPAN2* | tetraspanin 2 | EC Sp | PB |
| *TUBD1* | tubulin, delta 1 | Cyt | NB |
| *UBE2B* | ubiquitin-conjugating enzyme E2B | Cyt | Enzyme |
| *UIMC1* | ubiquitin interaction motif containing 1 | Nucl | PB |
| *VAV1* | vav 1 guanine nucleotide exchange factor | Nucl | TR |
| *ZBED2* | zinc finger, BED-type containing 2 | nc | NB |
| *ZBTB32* | zinc finger and BTB domain containing 32 | Nucl | TR |
| *ZC3H15* | zinc finger CCCH-type containing 15 | Nucl | PB |
| *ZHX2* | zinc fingers and homeoboxes 2 | Nucl | TR |
| *ZNF32* | zinc finger protein 32 | Nucl | NB |
| *ZSCAN16* | zinc finger and SCAN domain containing 16 | Nucl | TR |
