# Supplementary material for: Cross-platform comparison of independent datasets identifies an immune signature associated with improved survival in metastatic melanoma
Source: Oncotarget. 2016 Feb 13;7(12):14415–28. doi: 10.18632/oncotarget.7361 (PMC4924725; doi:10.18632/oncotarget.7361)
Supplement: Supplementary file 3 [file oncotarget-07-14415-s003.docx]

**Table S2: List of complete GO terms enriched in “favorable outcome signature”.** Output from Gene Ontology Consortium.

| GO Term | *Homo sapiens* - Background frequency | "Favorable Outcome Signature" frequency | Expected | (Over/under) | Fold Enrichment | P-value |
| --- | --- | --- | --- | --- | --- | --- |
| GO:0002376~immune system process | 2025 | 104 | 21.08 | + | 4.93 | 1.23E-42 |
| GO:0006955~immune response | 1227 | 74 | 12.77 | + | >5 | 3.86E-32 |
| GO:0002682~regulation of immune system process | 1166 | 66 | 12.14 | + | >5 | 2.19E-26 |
| GO:0006952~defense response | 1322 | 66 | 13.76 | + | 4.80 | 2.62E-23 |
| GO:0002684~positive regulation of immune system process | 746 | 50 | 7.77 | + | >5 | 5.51E-22 |
| GO:0045321~leukocyte activation | 401 | 37 | 4.18 | + | >5 | 7.15E-20 |
| GO:0050896~response to stimulus | 7080 | 146 | 73.71 | + | 1.98 | 6.99E-19 |
| GO:0050776~regulation of immune response | 742 | 46 | 7.73 | + | >5 | 1.53E-18 |
| GO:0051249~regulation of lymphocyte activation | 336 | 33 | 3.50 | + | >5 | 3.51E-18 |
| GO:0046649~lymphocyte activation | 329 | 32 | 3.43 | + | >5 | 2.06E-17 |
| GO:0050867~positive regulation of cell activation | 269 | 29 | 2.80 | + | >5 | 1.12E-16 |
| GO:0050863~regulation of T cell activation | 251 | 28 | 2.61 | + | >5 | 2.21E-16 |
| GO:0002694~regulation of leukocyte activation | 389 | 33 | 4.05 | + | >5 | 2.77E-16 |
| GO:0050865~regulation of cell activation | 420 | 34 | 4.37 | + | >5 | 2.96E-16 |
| GO:0006950~response to stress | 3211 | 90 | 33.43 | + | 2.69 | 3.86E-16 |
| GO:0001775~cell activation | 625 | 40 | 6.51 | + | >5 | 4.02E-16 |
| GO:1903037~regulation of leukocyte cell-cell adhesion | 257 | 28 | 2.68 | + | >5 | 4.06E-16 |
| GO:0007159~leukocyte adhesion | 258 | 28 | 2.69 | + | >5 | 4.49E-16 |
| GO:0002696~positive regulation of leukocyte activation | 258 | 28 | 2.69 | + | >5 | 4.49E-16 |
| GO:0051251~positive regulation of lymphocyte activation | 236 | 27 | 2.46 | + | >5 | 5.78E-16 |
| GO:0048583~regulation of response to stimulus | 3252 | 90 | 33.86 | + | 2.66 | 8.98E-16 |
| GO:0034110~regulation of homotypic cell-cell adhesion | 266 | 28 | 2.77 | + | >5 | 9.84E-16 |
| GO:0050851~antigen receptor-mediated signaling pathway | 120 | 21 | 1.25 | + | >5 | 2.01E-15 |
| GO:0050778~positive regulation of immune response | 482 | 35 | 5.02 | + | >5 | 2.31E-15 |
| GO:0070489~T cell aggregation | 220 | 25 | 2.29 | + | >5 | 1.68E-14 |
| GO:0042110~T cell activation | 220 | 25 | 2.29 | + | >5 | 1.68E-14 |
| GO:0071593~lymphocyte aggregation | 222 | 25 | 2.31 | + | >5 | 2.07E-14 |
| GO:0022407~regulation of cell-cell adhesion | 334 | 29 | 3.48 | + | >5 | 3.35E-14 |
| GO:0044700~single organism signaling | 4866 | 110 | 50.66 | + | 2.17 | 3.56E-14 |
| GO:0023052~signaling | 4866 | 110 | 50.66 | + | 2.17 | 3.56E-14 |
| GO:0070486~leukocyte aggregation | 228 | 25 | 2.37 | + | >5 | 3.81E-14 |
| GO:0007165~signal transduction | 4465 | 104 | 46.48 | + | 2.24 | 7.39E-14 |
| GO:0002429~immune response-activating cell surface receptor signaling pathway | 188 | 23 | 1.96 | + | >5 | 8.47E-14 |
| GO:0048518~positive regulation of biological process | 4724 | 107 | 49.18 | + | 2.18 | 1.33E-13 |
| GO:0048584~positive regulation of response to stimulus | 1696 | 60 | 17.66 | + | 3.40 | 1.88E-13 |
| GO:0007154~cell communication | 4975 | 110 | 51.79 | + | 2.12 | 1.94E-13 |
| GO:0034109~homotypic cell-cell adhesion | 272 | 26 | 2.83 | + | >5 | 2.07E-13 |
| GO:0016337~single organismal cell-cell adhesion | 435 | 31 | 4.53 | + | >5 | 4.98E-13 |
| GO:0050870~positive regulation of T cell activation | 182 | 22 | 1.90 | + | >5 | 5.67E-13 |
| GO:0034097~response to cytokine stimulus | 619 | 36 | 6.44 | + | >5 | 6.85E-13 |
| GO:1903039~positive regulation of leukocyte cell-cell adhesion | 184 | 22 | 1.92 | + | >5 | 7.09E-13 |
| GO:0034112~positive regulation of homotypic cell-cell adhesion | 186 | 22 | 1.94 | + | >5 | 8.83E-13 |
| GO:0022409~positive regulation of cell-cell adhesion | 217 | 23 | 2.26 | + | >5 | 1.77E-12 |
| GO:0098602~single organism cell adhesion | 457 | 31 | 4.76 | + | >5 | 1.89E-12 |
| GO:0065007~biological regulation | 10450 | 169 | 108.80 | + | 1.55 | 2.63E-12 |
| GO:0002768~immune response-regulating cell surface receptor signaling pathway | 307 | 26 | 3.20 | + | >5 | 3.52E-12 |
| GO:0071345~cellular response to cytokine stimulus | 517 | 32 | 5.38 | + | >5 | 7.53E-12 |
| GO:0030155~regulation of cell adhesion | 561 | 33 | 5.84 | + | >5 | 1.08E-11 |
| GO:0002757~immune response-activating signal transduction | 304 | 25 | 3.17 | + | >5 | 2.56E-11 |
| GO:0002764~immune response-regulating signal transduction | 410 | 28 | 4.27 | + | >5 | 4.98E-11 |
| GO:0050852~T cell receptor signaling pathway | 94 | 16 | 0.98 | + | >5 | 6.16E-11 |
| GO:0019221~cytokine-mediated signaling pathway | 383 | 27 | 3.99 | + | >5 | 7.40E-11 |
| GO:0002253~activation of immune response | 351 | 26 | 3.65 | + | >5 | 7.72E-11 |
| GO:0002252~immune effector process | 420 | 28 | 4.37 | + | >5 | 8.94E-11 |
| GO:0050794~regulation of cellular process | 9560 | 156 | 99.53 | + | 1.57 | 2.56E-10 |
| GO:0050789~regulation of biological process | 9962 | 160 | 103.70 | + | 1.54 | 2.72E-10 |
| GO:0051716~cellular response to stimulus | 5701 | 112 | 59.35 | + | 1.89 | 5.26E-10 |
| GO:0045087~innate immune response | 835 | 37 | 8.69 | + | 4.26 | 1.01E-09 |
| GO:0045785~positive regulation of cell adhesion | 338 | 24 | 3.52 | + | >5 | 2.10E-09 |
| GO:0048522~positive regulation of cellular process | 4038 | 89 | 42.04 | + | 2.12 | 2.23E-09 |
| GO:0044763~single-organism cellular process | 10911 | 167 | 113.60 | + | 1.47 | 2.98E-09 |
| GO:0006954~inflammatory response | 416 | 26 | 4.33 | + | >5 | 3.57E-09 |
| GO:0008150~biological_process | 15968 | 209 | 166.20 | + | 1.26 | 5.06E-09 |
| GO:0031295~T cell costimulation | 69 | 13 | 0.72 | + | >5 | 6.03E-09 |
| GO:0031294~lymphocyte costimulation | 70 | 13 | 0.73 | + | >5 | 7.21E-09 |
| GO:0007155~cell adhesion | 999 | 39 | 10.40 | + | 3.75 | 9.96E-09 |
| GO:0022610~biological adhesion | 1003 | 39 | 10.44 | + | 3.74 | 1.12E-08 |
| GO:0034341~response to interferon-gamma | 123 | 15 | 1.28 | + | >5 | 4.67E-08 |
| GO:0071346~cellular response to interferon-gamma | 103 | 14 | 1.07 | + | >5 | 5.83E-08 |
| GO:0044699~single-organism process | 12215 | 175 | 127.20 | + | 1.38 | 2.15E-07 |
| GO:0009607~response to biotic stimulus | 677 | 30 | 7.05 | + | 4.26 | 2.60E-07 |
| GO:0007166~cell surface receptor linked signal transduction | 2837 | 67 | 29.54 | + | 2.27 | 4.00E-07 |
| GO:0060333~interferon-gamma-mediated signaling pathway | 77 | 12 | 0.80 | + | >5 | 4.00E-07 |
| GO:0051707~response to other organism | 649 | 29 | 6.76 | + | 4.29 | 4.68E-07 |
| GO:0043207~response to external biotic stimulus | 649 | 29 | 6.76 | + | 4.29 | 4.68E-07 |
| GO:0030217~T cell differentiation | 123 | 14 | 1.28 | + | >5 | 5.83E-07 |
| GO:0009987~cellular process | 13503 | 184 | 140.60 | + | 1.31 | 2.66E-06 |
| GO:0031347~regulation of defense response | 525 | 25 | 5.47 | + | 4.57 | 2.99E-06 |
| GO:0010033~response to organic substance | 2305 | 57 | 24.00 | + | 2.38 | 3.30E-06 |
| GO:0009966~regulation of signal transduction | 2504 | 59 | 26.07 | + | 2.26 | 9.37E-06 |
| GO:0002443~leukocyte mediated immunity | 154 | 14 | 1.60 | + | >5 | 1.02E-05 |
| GO:0023051~regulation of signaling | 2793 | 63 | 29.08 | + | 2.17 | 1.19E-05 |
| GO:0010646~regulation of cell communication | 2804 | 63 | 29.19 | + | 2.16 | 1.40E-05 |
| GO:0019882~antigen processing and presentation | 224 | 16 | 2.33 | + | >5 | 2.04E-05 |
| GO:0048002~antigen processing and presentation of peptide antigen | 194 | 15 | 2.02 | + | >5 | 2.27E-05 |
| GO:0030098~lymphocyte differentiation | 198 | 15 | 2.06 | + | >5 | 2.98E-05 |
| GO:0050670~regulation of lymphocyte proliferation | 168 | 14 | 1.75 | + | >5 | 3.04E-05 |
| GO:0002449~lymphocyte mediated immunity | 114 | 12 | 1.19 | + | >5 | 3.17E-05 |
| GO:0032944~regulation of mononuclear cell proliferation | 169 | 14 | 1.76 | + | >5 | 3.28E-05 |
| GO:0002478~antigen processing and presentation of exogenous peptide antigen | 171 | 14 | 1.78 | + | >5 | 3.79E-05 |
| GO:0050900~leukocyte migration | 237 | 16 | 2.47 | + | >5 | 4.46E-05 |
| GO:0030097~hemopoiesis | 513 | 23 | 5.34 | + | 4.31 | 4.76E-05 |
| GO:0048534~hemopoietic or lymphoid organ development | 559 | 24 | 5.82 | + | 4.12 | 4.99E-05 |
| GO:0070663~regulation of leukocyte proliferation | 175 | 14 | 1.82 | + | >5 | 5.06E-05 |
| GO:0019884~antigen processing and presentation of exogenous antigen | 178 | 14 | 1.85 | + | >5 | 6.24E-05 |
| GO:0002521~leukocyte differentiation | 284 | 17 | 2.96 | + | >5 | 8.50E-05 |
| GO:0045619~regulation of lymphocyte differentiation | 125 | 12 | 1.30 | + | >5 | 8.66E-05 |
| GO:0032496~response to lipopolysaccharide | 250 | 16 | 2.60 | + | >5 | 9.30E-05 |
| GO:0051704~multi-organism process | 2259 | 53 | 23.52 | + | 2.25 | 9.72E-05 |
| GO:0002520~immune system development | 588 | 24 | 6.12 | + | 3.92 | 1.29E-04 |
| GO:0006959~humoral immune response | 131 | 12 | 1.36 | + | >5 | 1.44E-04 |
| GO:0035556~intracellular signal transduction | 1525 | 41 | 15.88 | + | 2.58 | 1.65E-04 |
| GO:0002237~response to molecule of bacterial origin | 263 | 16 | 2.74 | + | >5 | 1.86E-04 |
| GO:1902531~regulation of intracellular signal transduction | 1541 | 41 | 16.04 | + | 2.56 | 2.20E-04 |
| GO:0071310~cellular response to organic substance | 1728 | 44 | 17.99 | + | 2.45 | 2.23E-04 |
| GO:0009617~response to bacterium | 427 | 20 | 4.45 | + | 4.50 | 2.41E-04 |
| GO:0008219~cell death | 1089 | 33 | 11.34 | + | 2.91 | 3.17E-04 |
| GO:0006915~apoptosis | 1034 | 32 | 10.76 | + | 2.97 | 3.23E-04 |
| GO:0016265~death | 1094 | 33 | 11.39 | + | 2.90 | 3.53E-04 |
| GO:0050671~positive regulation of lymphocyte proliferation | 115 | 11 | 1.20 | + | >5 | 3.69E-04 |
| GO:0002697~regulation of immune effector process | 278 | 16 | 2.89 | + | >5 | 3.94E-04 |
| GO:0032946~positive regulation of mononuclear cell proliferation | 116 | 11 | 1.21 | + | >5 | 4.02E-04 |
| GO:0002460~adaptive immune response based on somatic recombination of immune receptors built from immunoglobulin superfamily domains | 118 | 11 | 1.23 | + | >5 | 4.76E-04 |
| GO:0012501~programmed cell death | 1054 | 32 | 10.97 | + | 2.92 | 4.99E-04 |
| GO:1902105~regulation of leukocyte differentiation | 211 | 14 | 2.20 | + | >5 | 5.00E-04 |
| GO:0070665~positive regulation of leukocyte proliferation | 120 | 11 | 1.25 | + | >5 | 5.63E-04 |
| GO:0002250~adaptive immune response | 152 | 12 | 1.58 | + | >5 | 7.09E-04 |
| GO:0045088~regulation of innate immune response | 254 | 15 | 2.64 | + | >5 | 7.49E-04 |
| GO:0050853~B cell receptor signaling pathway | 35 | 7 | 0.36 | + | >5 | 8.34E-04 |
| GO:0009893~positive regulation of metabolic process | 3010 | 61 | 31.34 | + | 1.95 | 1.23E-03 |
| GO:0009605~response to external stimulus | 1785 | 43 | 18.58 | + | 2.31 | 1.53E-03 |
| GO:0070887~cellular response to chemical stimulus | 2137 | 48 | 22.25 | + | 2.16 | 2.06E-03 |
| GO:1903706~regulation of hemopoiesis | 277 | 15 | 2.88 | + | >5 | 2.23E-03 |
| GO:0080134~regulation of response to stress | 1019 | 30 | 10.61 | + | 2.83 | 2.55E-03 |
| GO:0050871~positive regulation of B cell activation | 62 | 8 | 0.65 | + | >5 | 2.80E-03 |
| GO:0019222~regulation of metabolic process | 6091 | 98 | 63.41 | + | 1.55 | 4.72E-03 |
| GO:0023056~positive regulation of signaling | 1352 | 35 | 14.08 | + | 2.49 | 4.97E-03 |
| GO:0009967~positive regulation of signal transduction | 1234 | 33 | 12.85 | + | 2.57 | 5.33E-03 |
| GO:0019886~antigen processing and presentation of exogenous peptide antigen via MHC class II | 93 | 9 | 0.97 | + | >5 | 5.61E-03 |
| GO:0010647~positive regulation of cell communication | 1360 | 35 | 14.16 | + | 2.47 | 5.68E-03 |
| GO:0070098~chemokine-mediated signaling pathway | 47 | 7 | 0.49 | + | >5 | 5.91E-03 |
| GO:0032101~regulation of response to external stimulus | 625 | 22 | 6.51 | + | 3.38 | 6.24E-03 |
| GO:0050790~regulation of catalytic activity | 1953 | 44 | 20.33 | + | 2.16 | 6.67E-03 |
| GO:0002495~antigen processing and presentation of peptide antigen via MHC class II | 95 | 9 | 0.99 | + | >5 | 6.68E-03 |
| GO:0002274~myeloid leukocyte activation | 95 | 9 | 0.99 | + | >5 | 6.68E-03 |
| GO:0042129~regulation of T cell proliferation | 125 | 10 | 1.30 | + | >5 | 7.49E-03 |
| GO:0002504~antigen processing and presentation of peptide or polysaccharide antigen via MHC class II | 97 | 9 | 1.01 | + | >5 | 7.91E-03 |
| GO:0050864~regulation of B cell activation | 97 | 9 | 1.01 | + | >5 | 7.91E-03 |
| GO:0065009~regulation of molecular function | 2383 | 50 | 24.81 | + | 2.02 | 8.28E-03 |
| GO:0051239~regulation of multicellular organismal process | 2175 | 47 | 22.64 | + | 2.08 | 8.41E-03 |
| GO:0050868~negative regulation of T cell activation | 72 | 8 | 0.75 | + | >5 | 8.47E-03 |
| GO:0045580~regulation of T cell differentiation | 98 | 9 | 1.02 | + | >5 | 8.60E-03 |
| GO:0002706~regulation of lymphocyte mediated immunity | 98 | 9 | 1.02 | + | >5 | 8.60E-03 |
| GO:0002577~regulation of antigen processing and presentation | 17 | 5 | 0.18 | + | >5 | 8.83E-03 |
| GO:0001817~regulation of cytokine production | 490 | 19 | 5.10 | + | 3.72 | 9.02E-03 |
| GO:0051240~positive regulation of multicellular organismal process | 1204 | 32 | 12.53 | + | 2.55 | 9.13E-03 |
| GO:0002683~negative regulation of immune system process | 312 | 15 | 3.25 | + | 4.62 | 9.65E-03 |
| GO:0042221~response to chemical stimulus | 3591 | 66 | 37.39 | + | 1.77 | 1.13E-02 |
| GO:1903038~negative regulation of leukocyte cell-cell adhesion | 77 | 8 | 0.80 | + | >5 | 1.39E-02 |
| GO:0045058~T cell selection | 34 | 6 | 0.35 | + | >5 | 1.40E-02 |
| GO:0045061~thymic T cell selection | 19 | 5 | 0.20 | + | >5 | 1.51E-02 |
| GO:0007599~hemostasis | 508 | 19 | 5.29 | + | 3.59 | 1.52E-02 |
| GO:0050854~regulation of antigen receptor-mediated signaling pathway | 35 | 6 | 0.36 | + | >5 | 1.66E-02 |
| GO:0043368~positive T cell selection | 20 | 5 | 0.21 | + | >5 | 1.94E-02 |
| GO:0045059~positive thymic T cell selection | 9 | 4 | 0.09 | + | >5 | 2.14E-02 |
| GO:0051246~regulation of protein metabolic process | 2112 | 45 | 21.99 | + | 2.05 | 2.18E-02 |
| GO:0034111~negative regulation of homotypic cell-cell adhesion | 82 | 8 | 0.85 | + | >5 | 2.19E-02 |
| GO:0044093~positive regulation of molecular function | 1469 | 35 | 15.29 | + | 2.29 | 3.17E-02 |
| GO:0002377~immunoglobulin production | 40 | 6 | 0.42 | + | >5 | 3.53E-02 |
| GO:1902107~positive regulation of leukocyte differentiation | 118 | 9 | 1.23 | + | >5 | 3.82E-02 |
| GO:0060255~regulation of macromolecule metabolic process | 5194 | 84 | 54.07 | + | 1.55 | 4.51E-02 |
| GO:0016064~immunoglobulin mediated immune response | 68 | 7 | 0.71 | + | >5 | 6.52E-02 |
| GO:0042981~regulation of apoptosis | 1331 | 32 | 13.86 | + | 2.31 | 7.17E-02 |
| GO:0042127~regulation of cell proliferation | 1401 | 33 | 14.59 | + | 2.26 | 7.78E-02 |
| GO:0019724~B cell mediated immunity | 70 | 7 | 0.73 | + | >5 | 7.85E-02 |
| GO:0030595~leukocyte chemotaxis | 98 | 8 | 1.02 | + | >5 | 7.92E-02 |
| GO:0043067~regulation of programmed cell death | 1341 | 32 | 13.96 | + | 2.29 | 8.32E-02 |
| GO:0002503~peptide antigen assembly with MHC class II protein complex | 4 | 3 | 0.04 | + | >5 | 8.49E-02 |
| GO:0002399~MHC class II protein complex assembly | 4 | 3 | 0.04 | + | >5 | 8.49E-02 |
| GO:0050857~positive regulation of antigen receptor-mediated signaling pathway | 13 | 4 | 0.14 | + | >5 | 9.02E-02 |
| GO:0002703~regulation of leukocyte mediated immunity | 132 | 9 | 1.37 | + | >5 | 9.26E-02 |
| GO:1901700~response to oxygen-containing compound | 1222 | 30 | 12.72 | + | 2.36 | 9.45E-02 |
| GO:0051250~negative regulation of lymphocyte activation | 103 | 8 | 1.07 | + | >5 | 1.13E-01 |
| GO:0098542~defense response to other organism | 338 | 14 | 3.52 | + | 3.98 | 1.15E-01 |
| GO:0045621~positive regulation of lymphocyte differentiation | 75 | 7 | 0.78 | + | >5 | 1.22E-01 |
| GO:0002440~production of molecular mediator of immune response | 50 | 6 | 0.52 | + | >5 | 1.24E-01 |
| GO:0071216~cellular response to biotic stimulus | 137 | 9 | 1.43 | + | >5 | 1.24E-01 |
| GO:0031325~positive regulation of cellular metabolic process | 2485 | 48 | 25.87 | + | 1.86 | 1.33E-01 |
| GO:0032268~regulation of cellular protein metabolic process | 1916 | 40 | 19.95 | + | 2.01 | 1.38E-01 |
| GO:0033077~T cell differentiation in the thymus | 52 | 6 | 0.54 | + | >5 | 1.54E-01 |
| GO:0016477~cell migration | 713 | 21 | 7.42 | + | 2.83 | 1.64E-01 |
| GO:0002578~negative regulation of antigen processing and presentation | 5 | 3 | 0.05 | + | >5 | 1.65E-01 |
| GO:0002501~peptide antigen assembly with MHC protein complex | 5 | 3 | 0.05 | + | >5 | 1.65E-01 |
| GO:0002396~MHC protein complex assembly | 5 | 3 | 0.05 | + | >5 | 1.65E-01 |
| GO:0032729~positive regulation of interferon-gamma production | 53 | 6 | 0.55 | + | >5 | 1.71E-01 |
| GO:0010604~positive regulation of macromolecule metabolic process | 2365 | 46 | 24.62 | + | 1.87 | 1.74E-01 |
| GO:0008284~positive regulation of cell proliferation | 776 | 22 | 8.08 | + | 2.72 | 1.84E-01 |
| GO:0050817~coagulation | 503 | 17 | 5.24 | + | 3.25 | 1.91E-01 |
| GO:0007596~blood coagulation | 503 | 17 | 5.24 | + | 3.25 | 1.91E-01 |
| GO:0032649~regulation of interferon-gamma production | 81 | 7 | 0.84 | + | >5 | 1.98E-01 |
| GO:0045954~positive regulation of natural killer cell mediated cytotoxicity | 16 | 4 | 0.17 | + | >5 | 2.02E-01 |
| GO:0051674~localization of cell | 781 | 22 | 8.13 | + | 2.71 | 2.02E-01 |
| GO:0048870~cell motility | 781 | 22 | 8.13 | + | 2.71 | 2.02E-01 |
| GO:0071222~cellular response to lipopolysaccharide | 113 | 8 | 1.18 | + | >5 | 2.17E-01 |
| GO:0010941~regulation of cell death | 1409 | 32 | 14.67 | + | 2.18 | 2.19E-01 |
| GO:1903708~positive regulation of hemopoiesis | 148 | 9 | 1.54 | + | >5 | 2.25E-01 |
| GO:0042102~positive regulation of T cell proliferation | 83 | 7 | 0.86 | + | >5 | 2.31E-01 |
| GO:0031399~regulation of protein modification process | 1283 | 30 | 13.36 | + | 2.25 | 2.34E-01 |
| GO:0022408~negative regulation of cell-cell adhesion | 115 | 8 | 1.20 | + | >5 | 2.45E-01 |
| GO:0046651~lymphocyte proliferation | 84 | 7 | 0.87 | + | >5 | 2.49E-01 |
| GO:0006874~cellular calcium ion homeostasis | 273 | 12 | 2.84 | + | 4.22 | 2.67E-01 |
| GO:0043085~positive regulation of catalytic activity | 1228 | 29 | 12.78 | + | 2.27 | 2.69E-01 |
| GO:0060326~cell chemotaxis | 152 | 9 | 1.58 | + | >5 | 2.76E-01 |
| GO:0032943~mononuclear cell proliferation | 86 | 7 | 0.90 | + | >5 | 2.88E-01 |
| GO:0006968~cellular defense response | 59 | 6 | 0.61 | + | >5 | 3.09E-01 |
| GO:0065008~regulation of biological quality | 2871 | 52 | 29.89 | + | 1.74 | 3.13E-01 |
| GO:2000026~regulation of multicellular organismal development | 1370 | 31 | 14.26 | + | 2.17 | 3.16E-01 |
| GO:0040011~locomotion | 1175 | 28 | 12.23 | + | 2.29 | 3.17E-01 |
| GO:0002717~positive regulation of natural killer cell mediated immunity | 18 | 4 | 0.19 | + | >5 | 3.18E-01 |
| GO:0071219~cellular response to molecule of bacterial origin | 120 | 8 | 1.25 | + | >5 | 3.30E-01 |
| GO:0002695~negative regulation of leukocyte activation | 121 | 8 | 1.26 | + | >5 | 3.49E-01 |
| GO:0031400~negative regulation of protein modification process | 426 | 15 | 4.44 | + | 3.38 | 3.65E-01 |
| GO:0050878~regulation of body fluid levels | 642 | 19 | 6.68 | + | 2.84 | 3.82E-01 |
| GO:0002685~regulation of leukocyte migration | 123 | 8 | 1.28 | + | >5 | 3.91E-01 |
| GO:0010605~negative regulation of macromolecule metabolic process | 1938 | 39 | 20.18 | + | 1.93 | 3.98E-01 |
| GO:0030890~positive regulation of B cell proliferation | 38 | 5 | 0.40 | + | >5 | 4.13E-01 |
| GO:0055074~calcium ion homeostasis | 286 | 12 | 2.98 | + | 4.03 | 4.14E-01 |
| GO:0072503~cellular divalent inorganic cation homeostasis | 286 | 12 | 2.98 | + | 4.03 | 4.14E-01 |
| GO:0080090~regulation of primary metabolic process | 5159 | 80 | 53.71 | + | 1.49 | 4.16E-01 |
| GO:0070661~leukocyte proliferation | 92 | 7 | 0.96 | + | >5 | 4.39E-01 |
| GO:0050793~regulation of developmental process | 1951 | 39 | 20.31 | + | 1.92 | 4.59E-01 |
| GO:0001819~positive regulation of cytokine production | 336 | 13 | 3.50 | + | 3.72 | 4.61E-01 |
| GO:0044419~interspecies interaction between organisms | 709 | 20 | 7.38 | + | 2.71 | 4.65E-01 |
| GO:0044403~symbiosis, encompassing mutualism through parasitism | 709 | 20 | 7.38 | + | 2.71 | 4.65E-01 |
| GO:0008283~cell proliferation | 652 | 19 | 6.79 | + | 2.80 | 4.67E-01 |
| GO:1902533~positive regulation of intracellular signal transduction | 770 | 21 | 8.02 | + | 2.62 | 4.88E-01 |
| GO:0042113~B cell activation | 130 | 8 | 1.35 | + | >5 | 5.72E-01 |
| GO:0098751~bone cell development | 21 | 4 | 0.22 | + | >5 | 5.76E-01 |
| GO:0051247~positive regulation of protein metabolic process | 1217 | 28 | 12.67 | + | 2.21 | 5.78E-01 |
| GO:0001959~regulation of cytokine-mediated signaling pathway | 97 | 7 | 1.01 | + | >5 | 6.08E-01 |
| GO:0002583~regulation of antigen processing and presentation of peptide antigen | 8 | 3 | 0.08 | + | >5 | 6.58E-01 |
| GO:0002708~positive regulation of lymphocyte mediated immunity | 68 | 6 | 0.71 | + | >5 | 6.70E-01 |
| GO:0042269~regulation of natural killer cell mediated cytotoxicity | 22 | 4 | 0.23 | + | >5 | 6.88E-01 |
| GO:0050866~negative regulation of cell activation | 136 | 8 | 1.42 | + | >5 | 7.79E-01 |
| GO:0002715~regulation of natural killer cell mediated immunity | 23 | 4 | 0.24 | + | >5 | 8.15E-01 |
| GO:0042098~T cell proliferation | 44 | 5 | 0.46 | + | >5 | 8.17E-01 |
| GO:0072507~divalent inorganic cation homeostasis | 308 | 12 | 3.21 | + | 3.74 | 8.27E-01 |
| GO:0050920~regulation of chemotaxis | 138 | 8 | 1.44 | + | >5 | 8.60E-01 |
| GO:0060759~regulation of response to cytokine stimulus | 103 | 7 | 1.07 | + | >5 | 8.79E-01 |
| GO:0043066~negative regulation of apoptotic process | 804 | 21 | 8.37 | + | 2.51 | 8.87E-01 |
| GO:0032270~positive regulation of cellular protein metabolic process | 1119 | 26 | 11.65 | + | 2.23 | 9.06E-01 |
| GO:0032655~regulation of interleukin-12 production | 45 | 5 | 0.47 | + | >5 | 9.07E-01 |
| GO:0014066~regulation of phosphatidylinositol 3-kinase signaling | 72 | 6 | 0.75 | + | >5 | 9.12E-01 |
| GO:0033623~regulation of integrin activation | 9 | 3 | 0.09 | + | >5 | 9.30E-01 |
| GO:0060334~regulation of interferon-gamma-mediated signaling pathway | 24 | 4 | 0.25 | + | >5 | 9.58E-01 |
| GO:0002381~immunoglobulin production involved in immunoglobulin mediated immune response | 24 | 4 | 0.25 | + | >5 | 9.58E-01 |
| GO:0009612~response to mechanical stimulus | 180 | 9 | 1.87 | + | 4.80 | 9.84E-01 |
