# Supplementary material for: Cross-platform comparison of independent datasets identifies an immune signature associated with improved survival in metastatic melanoma
Source: Oncotarget. 2016 Feb 13;7(12):14415–28. doi: 10.18632/oncotarget.7361 (PMC4924725; doi:10.18632/oncotarget.7361)
Supplement: Supplementary file 4 [file oncotarget-07-14415-s004.docx]

**Table S3: Complete list of significant canonical pathways enriched in FOS.** Ingenuity Pathway Analysis also identified the FOS molecules for each pathway.

| Ingenuity Canonical Pathways | -log(p-value) | Ratio | Molecules |
| --- | --- | --- | --- |
| iCOS-iCOSL Signaling in T Helper Cells | 15.40 | 1.43E-01 | CD247, HLA-DMA, HLA-DOA, IL2RG, CD3E, HLA-DRB1, HLA-DQB1, NFKB1, CD3D, PTPRC, CD3G, LCK, CD40, HLA-DRA, HLA-DOB, VAV1, LCP2, IL2RB |
| Calcium-induced T Lymphocyte Apoptosis | 14.00 | 1.97E-01 | CD247, CD3G, HLA-DMA, LCK, HLA-DOA, CD3E, HLA-DRA, HLA-DOB, ATP2A3, HLA-DRB1, PRKCH, HLA-DQB1, CD3D, PRKCB |
| Type I Diabetes Mellitus Signaling | 12.90 | 1.32E-01 | CD247, HLA-DMA, HLA-DOA, CD3E, PIAS1, HLA-DRB1, HLA-DQB1, NFKB1, CD3D, IRF1, CD3G, HLA-DRA, HLA-DOB, STAT1, TNFRSF1B, FASLG |
| CD28 Signaling in T Helper Cells | 12.40 | 1.18E-01 | CD247, HLA-DMA, HLA-DOA, PTPN6, CD3E, HLA-DRB1, HLA-DQB1, NFKB1, CD3D, PTPRC, CD3G, LCK, HLA-DRA, HLA-DOB, VAV1, LCP2 |
| B Cell Development | 11.70 | 2.78E-01 | PTPRC, HLA-DMA, CD19, HLA-DOA, CD40, HLA-DRA, HLA-DRB1, HLA-DOB, HLA-DQB1, IL7 |
| Antigen Presentation Pathway | 11.00 | 2.38E-01 | PSMB9, HLA-DMA, HLA-DOA, HLA-DRA, HLA-DRB1, HLA-DOB, CD74, HLA-DPB1, TAP2, HLA-DPA1 |
| Primary Immunodeficiency Signaling | 10.80 | 1.72E-01 | BLNK, PTPRC, BTK, LCK, CD19, IL2RG, CD3E, CD40, CD8A, CD3D, TAP2 |
| Altered T Cell and B Cell Signaling in Rheumatoid Arthritis | 10.70 | 1.3E-01 | HLA-DMA, HLA-DOA, CD40, CXCL13, TNFSF13, HLA-DRA, TLR7, HLA-DOB, HLA-DRB1, HLA-DQB1, NFKB1, FASLG, TNFRSF17 |
| OX40 Signaling Pathway | 10.70 | 1.34E-01 | CD247, HLA-DMA, HLA-DOA, CD3E, HLA-DRB1, HLA-DQB1, NFKB1, CD3D, HLA-DPA1, CD3G, HLA-DRA, HLA-DOB, HLA-DPB1 |
| T Helper Cell Differentiation | 10.60 | 1.67E-01 | HLA-DMA, IL2RG, HLA-DOA, CD40, IL21R, HLA-DRA, IL10RA, HLA-DRB1, HLA-DOB, HLA-DQB1, TNFRSF1B, STAT1 |
| Crosstalk between Dendritic Cells and Natural Killer Cells | 10.40 | 1.23E-01 | IL15RA, IL2RG, CD40, KLRD1, KLRC4-KLRK1/KLRK1, HLA-DRA, TLR7, TNFSF10, HLA-DRB1, TNFRSF1B, NFKB1, FASLG, IL2RB |
| PKCθ Signaling in T Lymphocytes | 10.10 | 9.72E-02 | CD247, CD3G, HLA-DMA, LCK, HLA-DOA, CD3E, HLA-DRA, HLA-DOB, HLA-DRB1, VAV1, HLA-DQB1, NFKB1, CD3D, LCP2 |
| T Cell Receptor Signaling | 10.00 | 1.19E-01 | BTK, PTPRC, CD247, CD3G, LCK, CD3E, RASGRP1, VAV1, NFKB1, CD8A, CD8B, CD3D, LCP2 |
| Role of NFAT in Regulation of the Immune Response | 9.78 | 8E-02 | CD247, BLNK, HLA-DMA, HLA-DOA, CD3E, HLA-DRB1, HLA-DQB1, NFKB1, CD3D, BTK, CD3G, LCK, HLA-DRA, HLA-DOB, IKBKAP, LCP2 |
| Nur77 Signaling in T Lymphocytes | 9.16 | 1.56E-01 | CD247, CD3G, HLA-DMA, HLA-DOA, CD3E, HLA-DRA, HLA-DRB1, HLA-DOB, HLA-DQB1, CD3D |
| Cdc42 Signaling | 8.21 | 7.53E-02 | CD247, HLA-DMA, HLA-DOA, CD3E, HLA-DRB1, HLA-DQB1, CD3D, CLIP1, HLA-DPA1, CD3G, HLA-DRA, HLA-DOB, VAV1, HLA-DPB1 |
| Allograft Rejection Signaling | 7.36 | 1.03E-01 | HLA-DMA, HLA-DOA, CD40, HLA-DRA, HLA-DRB1, HLA-DOB, HLA-DQB1, HLA-DPB1, HLA-DPA1, FASLG |
| Autoimmune Thyroid Disease Signaling | 7.2 | 1.29E-01 | HLA-DMA, HLA-DOA, CD40, HLA-DRA, HLA-DRB1, HLA-DOB, HLA-DQB1, FASLG |
| Communication between Innate and Adaptive Immune Cells | 6.98 | 8.93E-02 | CCL4, CD40, TNFSF13, HLA-DRA, TLR7, HLA-DRB1, CCL5, CD8A, CD8B, TNFRSF17 |
| CCR5 Signaling in Macrophages | 6.93 | 9.28E-02 | CD247, CD3G, CCL4, CD3E, PRKCH, CCL5, CD3D, FASLG, PRKCB |
| Dendritic Cell Maturation | 6.76 | 6.16E-02 | HLA-DMA, HLA-DOA, IL32, HLA-DRB1, HLA-DQB1, NFKB1, CD40, HLA-DRA, HLA-DOB, LY75, IRF8, STAT1, TNFRSF1B |
| IL-4 Signaling | 6.72 | 1.12E-01 | RPS6KB1, HLA-DMA, IL2RG, PTPN6, HLA-DOA, HLA-DRA, HLA-DRB1, HLA-DOB, HLA-DQB1 |
| Natural Killer Cell Signaling | 6.2 | 8.47E-02 | CD247, LCK, PTPN6, SH2D1A, KLRD1, KLRC4-KLRK1/KLRK1, VAV1, PRKCH, LCP2, PRKCB |
| Phospholipase C Signaling | 6.18 | 5.28E-02 | BLNK, CD247, CD3E, NFKB1, CD3D, BTK, CD3G, LCK, PLA2G2D, PRKCH, RHOF, LCP2, FNBP1, PRKCB |
| CTLA4 Signaling in Cytotoxic T Lymphocytes | 6.07 | 9.38E-02 | CD247, CD3G, LCK, PTPN6, CD3E, CD8A, CD3D, CD8B, LCP2 |
| Graft-versus-Host Disease Signaling | 6.02 | 1.37E-01 | HLA-DMA, HLA-DOA, HLA-DRA, HLA-DRB1, HLA-DOB, HLA-DQB1, FASLG |
| Hematopoiesis from Pluripotent Stem Cells | 5.71 | 1.11E-01 | CD247, CD3G, CD3E, CD8A, CD3D, CD8B, IL7 |
| Pathogenesis of Multiple Sclerosis | 5.67 | 4E-01 | CXCL9, CCL4, CXCR3, CCL5 |
| Tec Kinase Signaling | 5.66 | 5.98E-02 | BTK, LCK, TNFSF10, VAV1, PRKCH, STAT1, RHOF, NFKB1, FNBP1, FASLG, PRKCB |
| Role of JAK1 and JAK3 in γc Cytokine Signaling | 5.12 | 1.03E-01 | BLNK, IL2RG, IL15RA, IL21R, STAT1, IL7, IL2RB |
| Cytotoxic T Lymphocyte-mediated Apoptosis of Target Cells | 4.5 | 1.28E-01 | CD247, CD3G, CD3E, CD3D, FASLG |
| Hepatic Fibrosis / Hepatic Stellate Cell Activation | 4.43 | 5.81E-02 | CXCL9, CD40, IL10RA, CXCR3, CCL5, TNFRSF1B, STAT1, NFKB1, FASLG |
| Production of Nitric Oxide and Reactive Oxygen Species in Macrophages | 4.31 | 4.72E-02 | PTPN6, PRKCH, IRF8, TNFRSF1B, STAT1, RHOF, NFKB1, FNBP1, IRF1, PRKCB |
| Induction of Apoptosis by HIV1 | 4.2 | 8.96E-02 | CXCR4, IKBKAP, TNFRSF1B, NFKB1, NAIP, FASLG |
| Atherosclerosis Signaling | 4.04 | 5.76E-02 | PLA2G2D, SELP, CD40, CXCR4, RARRES3, ALOX5, NFKB1, SELPLG |
| B Cell Receptor Signaling | 3.98 | 5.14E-02 | BLNK, PTPRC, BTK, RPS6KB1, CD19, PTPN6, VAV1, NFKB1, PRKCB |
| PI3K Signaling in B Lymphocytes | 3.89 | 5.59E-02 | BLNK, PTPRC, BTK, CD19, CD40, VAV1, NFKB1, PRKCB |
| Role of JAK1, JAK2 and TYK2 in Interferon Signaling | 3.8 | 1.43E-01 | PTPN6, PTPN2, STAT1, NFKB1 |
| IL-12 Signaling and Production in Macrophages | 3.75 | 5.1E-02 | CD40, MST1, PRKCH, IRF8, STAT1, NFKB1, IRF1, PRKCB |
| Systemic Lupus Erythematosus Signaling | 3.55 | 3.92E-02 | PTPRC, CD247, CD3G, LCK, PTPN6, CD3E, CD40, CD72, TLR7, CD3D |
| Regulation of IL-2 Expression in Activated and Anergic T Lymphocytes | 3.5 | 6.74E-02 | CD247, CD3G, CD3E, VAV1, NFKB1, CD3D |
| Death Receptor Signaling | 3.25 | 7.35E-02 | TNFSF10, TNFRSF1B, NFKB1, NAIP, FASLG |
| Interferon Signaling | 3.2 | 1.11E-01 | PTPN2, PIAS1, STAT1, IRF1 |
| IL-15 Signaling | 3.15 | 6.94E-02 | LCK, IL2RG, IL15RA, NFKB1, IL2RB |
| Fcγ Receptor-mediated Phagocytosis in Macrophages and Monocytes | 3.12 | 5.66E-02 | RPS6KB1, VAV1, PRKCH, FYB, LCP2, PRKCB |
| Eicosanoid Signaling | 3.08 | 5.81E-02 | LTA4H, PLA2G2D, RARRES3, ALOX5, PTGER4 |
| Role of Pattern Recognition Receptors in Recognition of Bacteria and Viruses | 3.05 | 5.5E-02 | CLEC7A, TLR7, PRKCH, CCL5, NFKB1, PRKCB |
| Granulocyte Adhesion and Diapedesis | 3 | 4.4E-02 | CXCL9, CCL4, SELP, CXCL13, CXCR4, CCL5, TNFRSF1B, SELPLG |
| Erythropoietin Signaling | 2.96 | 6.33E-02 | RPS6KB1, PTPN6, PRKCH, NFKB1, PRKCB |
| Growth Hormone Signaling | 2.9 | 6.41E-02 | RPS6KB1, PTPN6, PRKCH, STAT1, PRKCB |
| NF-κB Activation by Viruses | 2.77 | 6.02E-02 | LCK, IKBKAP, PRKCH, NFKB1, PRKCB |
| Fc Epsilon RI Signaling | 2.76 | 5.13E-02 | BTK, PLA2G2D, VAV1, PRKCH, LCP2, PRKCB |
| Renin-Angiotensin Signaling | 2.76 | 4.76E-02 | PTPN6, PRKCH, CCL5, STAT1, NFKB1, PRKCB |
| Type II Diabetes Mellitus Signaling | 2.62 | 3.51E-02 | SLC27A2, ACSL5, PRKCH, TNFRSF1B, NFKB1, PRKCB |
| p38 MAPK Signaling | 2.59 | 5E-02 | RPS6KB1, PLA2G2D, MAP4K1, TNFRSF1B, STAT1, FASLG |
| p70S6K Signaling | 2.55 | 4.55E-02 | BTK, RPS6KB1, CD19, IL2RG, PRKCH, PRKCB |
| IL-15 Production | 2.43 | 9.68E-02 | STAT1, NFKB1, IRF1 |
| TNFR2 Signaling | 2.39 | 8.82E-02 | TNFRSF1B, NFKB1, NAIP |
| NF-κB Signaling | 2.38 | 3.87E-02 | LCK, CD40, TLR7, TNFRSF1B, NFKB1, TNFRSF17, PRKCB |
| 4-1BB Signaling in T Lymphocytes | 2.26 | 8.33E-02 | TNFRSF9, IKBKAP, NFKB1 |
| 3-phosphoinositide Degradation | 2.22 | 3.7E-02 | PTPRC, INPP4A, PTPN6, PTPN2, PPP1R16B, DUSP2 |
| Agranulocyte Adhesion and Diapedesis | 2.22 | 3.65E-02 | CXCL9, CCL4, SELP, CXCL13, CXCR4, CCL5, SELPLG |
| Activation of IRF by Cytosolic Pattern Recognition Receptors | 2.21 | 5.48E-02 | CD40, IKBKAP, STAT1, NFKB1 |
| IL-8 Signaling | 2.19 | 3.11E-02 | RPS6KB1, CCND2, PRKCH, RHOF, NFKB1, FNBP1, PRKCB |
| MIF-mediated Glucocorticoid Regulation | 2.18 | 7.14E-02 | PLA2G2D, CD74, NFKB1 |
| Molecular Mechanisms of Cancer | 2.16 | 2.58E-02 | TCF4, CCND2, RASGRP1, PRKCH, RHOF, NFKB1, NAIP, FNBP1, FASLG, PRKCB |
| IL-9 Signaling | 2.15 | 7.5E-02 | IL2RG, STAT1, NFKB1 |
| Gαq Signaling | 2.11 | 3.51E-02 | BTK, PRKCH, RHOF, NFKB1, FNBP1, PRKCB |
| Chemokine Signaling | 2.07 | 5.33E-02 | CCL4, CXCR4, CCL5, PRKCB |
| Leukocyte Extravasation Signaling | 2.06 | 3.33E-02 | BTK, CXCR4, RASGRP1, VAV1, PRKCH, SELPLG, PRKCB |
| IL-3 Signaling | 2.02 | 5.33E-02 | PTPN6, PRKCH, STAT1, PRKCB |
| Role of Macrophages, Fibroblasts and Endothelial Cells in Rheumatoid Arthritis | 2.01 | 2.63E-02 | TCF4, IL32, TLR7, PRKCH, CCL5, TNFRSF1B, NFKB1, IL7, PRKCB |
| Fatty Acid Activation | 2.01 | 1.05E-01 | SLC27A2, ACSL5 |
| April Mediated Signaling | 2.01 | 6.82E-02 | TNFSF13, NFKB1, TNFRSF17 |
| Prolactin Signaling | 1.98 | 4.76E-02 | PRKCH, STAT1, IRF1, PRKCB |
| Phenylalanine Degradation IV (Mammalian, via Side Chain) | 1.95 | 5.13E-02 | ALDH2, SLC27A2 |
| Role of PKR in Interferon Induction and Antiviral Response | 1.95 | 6.12E-02 | STAT1, NFKB1, IRF1 |
| B Cell Activating Factor Signaling | 1.95 | 6.52E-02 | IKBKAP, NFKB1, TNFRSF17 |
| TREM1 Signaling | 1.94 | 4.44E-02 | CD40, TLR7, NFKB1, NAIP |
| Glucocorticoid Receptor Signaling | 1.93 | 2.68E-02 | CD247, CD3G, CD3E, TAF7, CCL5, STAT1, NFKB1, CD3D |
| MIF Regulation of Innate Immunity | 1.92 | 5.77E-02 | PLA2G2D, CD74, NFKB1 |
| Acute Myeloid Leukemia Signaling | 1.9 | 4.76E-02 | RPS6KB1, TCF4, PIM1, NFKB1 |
| Leukotriene Biosynthesis | 1.89 | 7.69E-02 | LTA4H, ALOX5 |
| VDR/RXR Activation | 1.89 | 4.55E-02 | PRKCH, CCL5, PSMC5, PRKCB |
| iNOS Signaling | 1.84 | 5.66E-02 | STAT1, NFKB1, IRF1 |
| D-myo-inositol (1,4,5,6)-Tetrakisphosphate Biosynthesis | 1.84 | 3.47E-02 | PTPRC, PTPN6, PTPN2, PPP1R16B, DUSP2 |
| D-myo-inositol (3,4,5,6)-tetrakisphosphate Biosynthesis | 1.84 | 3.47E-02 | PTPRC, PTPN6, PTPN2, PPP1R16B, DUSP2 |
| γ-linolenate Biosynthesis II (Animals) | 1.79 | 8.33E-02 | SLC27A2, ACSL5 |
| Mitochondrial L-carnitine Shuttle Pathway | 1.79 | 9.09E-02 | SLC27A2, ACSL5 |
| Differential Regulation of Cytokine Production in Macrophages and T Helper Cells by IL-17A and IL-17F | 1.74 | 1.11E-01 | CCL4, CCL5 |
| Tryptophan Degradation X (Mammalian, via Tryptamine) | 1.74 | 6.9E-02 | ALDH2, AKR1A1 |
| Apoptosis Signaling | 1.71 | 4E-02 | TNFRSF1B, NFKB1, NAIP, FASLG |
| CD27 Signaling in Lymphocytes | 1.67 | 5.08E-02 | IKBKAP, CD27, NFKB1 |
| HMGB1 Signaling | 1.63 | 3.67E-02 | TNFRSF1B, RHOF, NFKB1, FNBP1 |
| Colorectal Cancer Metastasis Signaling | 1.63 | 2.61E-02 | TCF4, TLR7, STAT1, RHOF, NFKB1, PTGER4, FNBP1 |
| UVB-Induced MAPK Signaling | 1.62 | 5.17E-02 | RPS6KB1, PRKCH, PRKCB |
| IL-2 Signaling | 1.62 | 4.92E-02 | LCK, IL2RG, IL2RB |
| D-myo-inositol-5-phosphate Metabolism | 1.62 | 3.09E-02 | PTPRC, PTPN6, PTPN2, PPP1R16B, DUSP2 |
| Thrombin Signaling | 1.61 | 2.84E-02 | RPS6KB1, PRKCH, RHOF, NFKB1, FNBP1, PRKCB |
| Thrombopoietin Signaling | 1.58 | 4.69E-02 | PRKCH, STAT1, PRKCB |
| Polyamine Regulation in Colon Cancer | 1.57 | 6.67E-02 | PSME1, TCF4 |
| Differential Regulation of Cytokine Production in Intestinal Epithelial Cells by IL-17A and IL-17F | 1.54 | 8.7E-02 | CCL4, CCL5 |
| Cholecystokinin/Gastrin-mediated Signaling | 1.52 | 3.77E-02 | PRKCH, RHOF, FNBP1, PRKCB |
| 3-phosphoinositide Biosynthesis | 1.49 | 2.76E-02 | PTPRC, PTPN6, PTPN2, PPP1R16B, DUSP2 |
| Rac Signaling | 1.48 | 3.12E-02 | RPS6KB1, CYFIP2, CYFIP1, NFKB1 |
| CXCR4 Signaling | 1.48 | 2.87E-02 | CXCR4, PRKCH, RHOF, FNBP1, PRKCB |
| IL-17A Signaling in Gastric Cells | 1.47 | 7.14E-02 | CCL5, NFKB1 |
| Tetrahydrobiopterin Biosynthesis I | 1.46 | 9.09E-02 | GCH1 |
| Tetrahydrobiopterin Biosynthesis II | 1.46 | 7.69E-02 | GCH1 |
| Protein Kinase A Signaling | 1.45 | 2.2E-02 | PTPRC, TCF4, PTPN6, PHKB, PTPN2, PRKCH, NFKB1, DUSP2, PRKCB |
| CD40 Signaling | 1.43 | 4.23E-02 | CD40, IKBKAP, NFKB1 |
| GM-CSF Signaling | 1.43 | 4.41E-02 | PIM1, STAT1, PRKCB |
| JAK/Stat Signaling | 1.36 | 4.23E-02 | PTPN6, PIAS1, STAT1 |
| Role of MAPK Signaling in the Pathogenesis of Influenza | 1.35 | 4.17E-02 | PLA2G2D, RARRES3, CCL5 |
| Phenylethylamine Degradation I | 1.34 | 9.09E-02 | ALDH2 |
| D-glucuronate Degradation I | 1.34 | 7.14E-02 | AKR1A1 |
| Fatty Acid β-oxidation I | 1.32 | 4.44E-02 | SLC27A2, ACSL5 |
